# Quality of Life, Safety and Efficacy Profile of Thermostable Flolan in Pulmonary Arterial Hypertension

**DOI:** 10.1371/journal.pone.0120657

**Published:** 2015-03-20

**Authors:** Steeve Provencher, Patrap Paruchuru, Andrea Spezzi, Brian Waterhouse, Mardi Gomberg-Maitland, on behalf of the pH12 Flolan reformulation study group

**Affiliations:** 1 Pulmonary Hypertension Research Group, Centre de recherche de l’Institut Universitaire de cardiologie et de pneumologie de Québec, Université Laval, Québec, Québec, Canada; 2 GlaxoSmithKline R&D, 980 Great West Road, Brentford, United Kingdom; 3 University of Chicago Medical Centre, 5841 S Maryland Ave, MC5403, Rm L08, Chicago, Illinois, United States of America; Kurume University School of Medicine, JAPAN

## Abstract

**Background:**

Flolan (epoprostenol sodium) is most commonly prescribed to patients with severe pulmonary arterial hypertension (PAH) owing to the requirement that the drug be delivered by continuous intravenous infusion and the reconstituted solution may only be administered up to 24 hours when it is maintained between a temperature of 2°C and 8°C. The aim of this single-arm, open label study was to describe the effects of the new thermostable formulation of Flolan on health-related quality of life (HRQoL) and ease of administration in subjects switching from the currently marketed Flolan to the reformulated product.

**Methods:**

Following a 4-week run-in period and after 4 weeks of treatment with the reformulated product, patients completed the SF-36 HRQoL questionnaire and a study-specific questionnaire evaluating ease of administration, along with World Health Organization (WHO) functional class, six-minute walked distance (6MWD) and N-terminal-pro B-type natriuretic peptide (NT-proBNP) assessment.

**Results:**

16 participants completed the study. The SF-36 scores remained unchanged from baseline to Week 4. Conversely, there were small improvements for the majority of the study-specific questionnaire items and 14 (88%) subjects preferred the reformulated product to the currently marketed Flolan. There was no significant change in the dose of reformulated product, 6MWD, Borg dyspnoea index, WHO functional class and mean NT-proBNP levels. No significant changes in haemodynamic parameters were seen from baseline to 2 hours post transition in a subset of patients undergoing catheterization.

**Conclusion:**

The reformulated product was not associated with significant improvement in HRQoL compared with the currently marketed Flolan as measured by the SF-36. However, most subjects preferred the reformulated product to the currently marketed Flolan. Moreover, the 2 formulations of Flolan had similar safety and efficacy profiles.

**Trial Registration:**

ClinicalTrials.gov NCT01462565

## Introduction

Pulmonary arterial hypertension (PAH) is a chronic debilitating disease characterised by a progressive increase of pulmonary vascular resistance leading to right ventricular failure and premature death [1]. PAH is associated with very poor quality of life similar to that of devastating conditions such as metastatic cancer [2–3], and carries an extremely poor prognosis in untreated patients, with a median survival of approximately 2 years [4]. The goals of therapy include improvement of symptoms, health-related quality of life (HRQoL) and prevention of progression of the disease. Current treatments include prostanoids, phosphodiesterase type 5 inhibitors and endothelin receptor antagonists [5].

Epoprostenol is the longest established therapy and the only approved therapy that has been clearly demonstrated to reduce mortality in idiopathic PAH [6]. Flolan (epoprostenol sodium), the synthetic form of the natural prostaglandin derivative prostacyclin (prostaglandin I2), is registered worldwide for the treatment of PAH. In clinical practice, epoprostenol is most commonly prescribed to patients with severe PAH owing to the requirement that the drug be delivered by continuous intravenous infusion due to its instability in solution and rapid metabolism *in vivo* [7]. However, Flolan requires reconstitution with sterile diluent, and the reconstituted solution may only be administered up to 24 hours when it is maintained between a temperature of 2°C and 8°C during infusion, thereby, necessitating the use of a cold pack. GlaxoSmithKline has recently reformulated the diluent for Flolan by increasing the pH of the diluent from 10.5 to 12.0. The reformulated product may be reconstituted and diluted every 6 days and is stable for 24 hours up to 35°C. Freshly prepared solutions of the reformulated product can be administered immediately or stored for up to 8 days at 2°C to 8°C prior to administration. Following this preparation or storage, the solution for infusion should be used within 72 hours at up to 25°C, 48 hours at up to 30°C, 24 hours at up to 35°C or 12 hours at up to 40°C. The reformulated product is thus anticipated to provide an added level of convenience to patients through reduction in the frequency of reconstitution, and elimination of the need for a cold pack.

The aim of this single-arm, open label study was to describe the effects of the new reformulated Flolan on HRQoL and ease of administration, and to determine the dose titration requirement in subjects switching from the currently marketed Flolan to the reformulated product.

## Materials and Methods

The protocol for this trial and supporting CONSORT checklist are available as supporting information; see S1 CONSORT Checklist and S1 Protocol.

### Patients

This was a multicentre, open label, single-arm study of 4 weeks’ duration in adult subjects who were receiving Flolan for the treatment of PAH. Therefore, all subjects received the reformulated product during the treatment period. Eligible patients included men or non-pregnant, non-lactating women aged 18 to 75 years who had been on a stable dose of Flolan for at least 3 months for the treatment of PAH prior to screening, and were on stable doses of other PAH treatments for at least 30 days prior to screening. Subjects were required to be able to walk 150 metres during a six-minute walking test. Subjects with a resting arterial oxygen saturation <90%, with congestive heart failure arising from severe left ventricular dysfunction, who had been hospitalised or had visited the emergency room for a PAH-related condition in the past 3 months, who were not expected to be clinically stable for the duration of the study, or who were taking Flolan for a condition or in a manner outside the approved indication were excluded.

### Ethics Statement

The study was approved by the ethics committees of the study centres (Institut universitaire de cardiologie et de pneumologie de Quebec, Canada; METC VU Medisch Centrum, Amsterdam, The Netherlands; Mt Sinai Medical Center, Miami Beach; Boston University Medical Center, Boston; Johns Hopkins University, Baltimore; and University of Chicago Hospitals, Chicago, USA) and was conducted according to the principles of the Declaration of Helsinki and good clinical practice. All patients gave written informed consent prior to study participation. All subjects were given the option to continue on the reformulated product until complete enrolment of all subjects. Initially this was thought to continue until FDA approval. However GlaxoSmithKline terminated the study because of the need for a more suitable storage container for the reformulated product.

### Procedures

Following a 4-week run-in period, eligible subjects were admitted to the clinic for baseline assessments and for switching to the reformulated product (study medication). Subjects remained in hospital for a minimum of 6 hours to ensure clinical and haemodynamic stability prior to discharge. Open-label supplies were provided to sites/subjects through a third party distributor in the US and Canada and through the hospital pharmacy and local city pharmacy in The Netherlands. Study drug was prepared by the site staff (or subject) by reconstituting and diluting one or more vials of epoprostenol lyophile (according to therapeutic need) with two vials of sterile glycine diluent (pH 12), to give 100 mL of medication for each day of treatment. This reconstituted infusion was transferred to medication cassettes prior to either refrigerated storage or immediate continuous intravenous infusion via a central venous catheter using an ambulatory infusion pump. Initially, the study drug was started at an equivalent dose to the subject’s currently marketed Flolan treatment. If necessary, the investigator adjusted the dose, as appropriate for the subject, until they were on a stable dose of the study drug.

Both at baseline (before the transition to the reformulated product) and after 4 weeks of treatment with the study drug, patients completed the Short Form 36 (SF-36) quality of life questionnaire [8] and a study-specific questionnaire evaluating ease of administration (**S1 QoLassessment**) along with an investigator assessment of World Health Organization (WHO) functional class. SF-36 scores at baseline and week 4 were summarised descriptively for each domain and component score. N-terminal-pro B-type natriuretic peptide (NT-proBNP, by Electro Chemiluminescense Immuno Assay (ECLIA)) was also measured at rest. Patients then performed a six-minute walking test according to current recommendations [9]. Breathlessness after the walking test was assessed using the Borg dyspnea index [10]. Finally, a subset of subjects had a right heart catheterisation in those sites where this was considered standard of care per local practice and at the discretion of the investigator. Haemodynamic parameters were collected in this subset at baseline (immediately prior to the switch to study drug) and at a single time point between 1 to 2 hours after the switch to study drug. All patients completed the treatment phase of the study with the first subject screened on 23rd November 2011 and the last subject completing the final visit of the treatment period on 16^th^ May 2012.

The primary efficacy endpoints for this study included: 1) HRQoL assessment using the SF-36 questionnaire; 2) Ease of administration and changes in activities of daily living assessment using a study-specific questionnaire; 3) Change from baseline in the dose of reformulated product. The secondary efficacy endpoints for this study were: 1) six-minute walked distance (6MWD) after 4 weeks of treatment; 2) breathlessness after 6MWD assessed using the Borg dyspnea index and; 3) WHO functional class at baseline and after 4 weeks of treatment.

### Statistical analysis

The results are presented as mean (standard deviation) unless otherwise specified. The sample size calculation based on feasibility, planned to enrol approximately 20 subjects in order to have at least 15 subjects completing the main part of the study. No imputation was made for missing data. All efficacy and safety analyses were based on the Intent-to-Treat population, which consisted of all subjects who received at least 1 dose of Flolan (either currently marketed Flolan during the run-in period or study drug during the treatment period). However, the 6MWD assessment was performed incorrectly for 2 subjects (subjects 090809/081 and 090809/082): they were asked to walk for 150 m rather than the required 6 minutes. Therefore, the 6MWD analysis excluded these 2 subjects. Endpoints were used on a descriptive basis without formal statistical comparison between visits.

## Results

### Patients’ disposition

**Fig 1:**
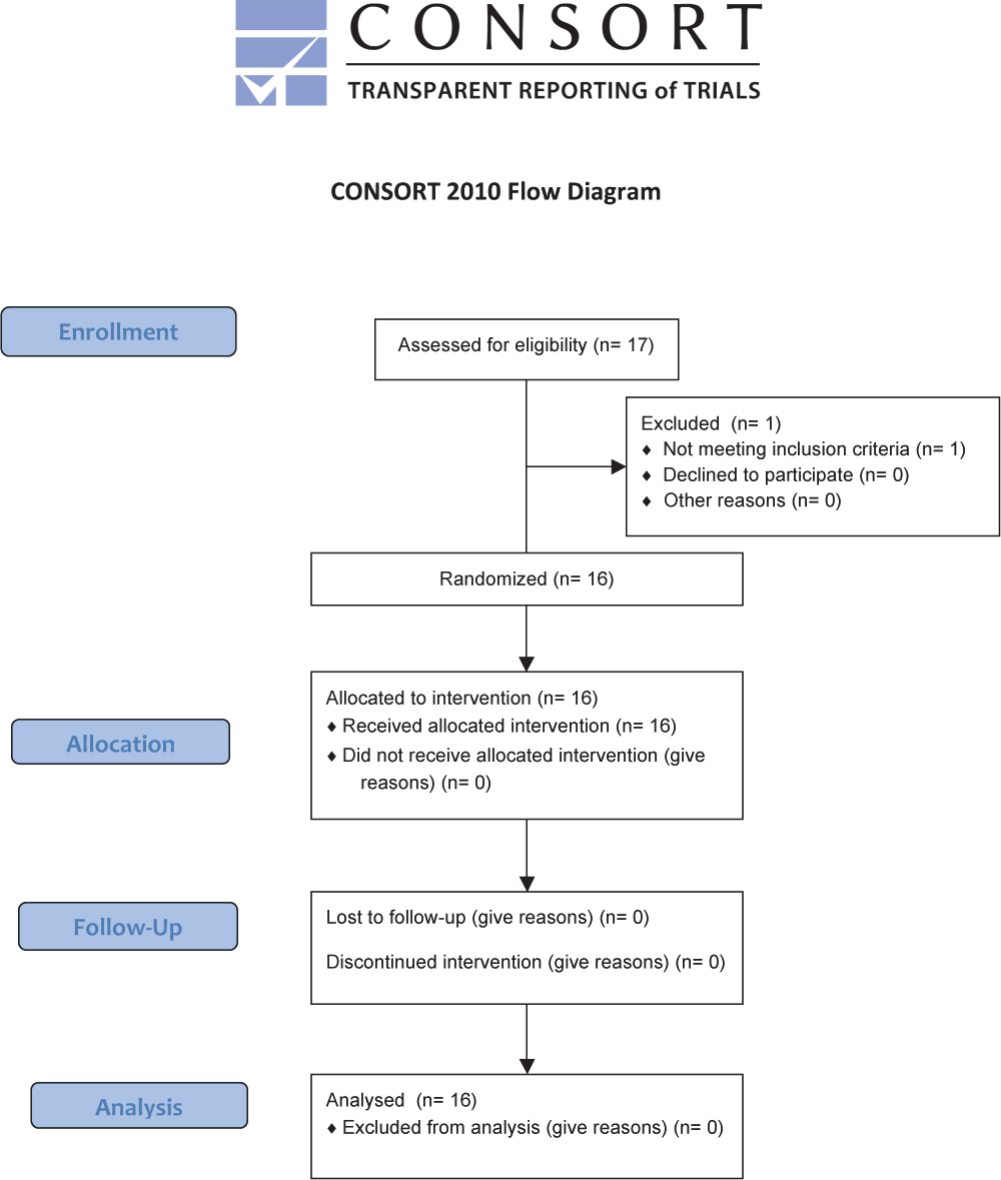
Study flow chart. One patient was excluded because of a catheter site infection which developed during screening period (before the baseline visit).

Of 17 subjects screened, 16 were enrolled from 7 study centres in the US (5 centres), Canada (1 centre) and the Netherlands (1 centre) (**Fig. 1**). Most patients were female with WHO functional class II-III idiopathic PAH and the median time since diagnosis was 4.2 years (range 0.5 years- 18.3 years) (**Table 1**). Fifteen subjects continued in to the extension phase. Initially this was thought to continue until regulatory approval, however GlaxoSmithKline terminated the study because of the need for a more suitable storage container for the reformulated product. The last subject was withdrawn from the study on 08^th^ November 2012.

**Table 1:** Baseline Characteristics of the study population.

Parameters	
Age, years, Mean (SD)	50.0 (13.4)
Sex, F/M	12/4
Caucasian/Hispanic	15/1
Body mass index, kg/m2, Mean (SD)	28 (6)
Time from diagnosis, years	
Mean, (SD)	6.1 (4.9)
Median (range)	4.2 (0.5–18.3)
Aetiology of PAH, n (%)	
Idiopathic PAH	9 (56)
Familial PAH	3 (19)
PAH associated with concomitant conditions	4 (25)
Hemodynammics, Mean (SD)*	
mRAP, mmHg	7.5 (5.1)
mPAP, mmHg	47.2 (18.9)
PCWP, mmHg	11.0 (4.2)
CI, L/min/Sq.m	2.9 (0.8)
PVR, mmHg/L/min	8.4 (5.5)
6MWD, meters, Mean (SD)	439 (100)
WHO FC	
II	10
III	6
Flolan dosage, ng/kg/min, Mean (SD)	36 (16)
Other therapies, n (%)	
Sildenafil	6 (38%)
Ambrisentan	3 (19%)
Warfarin	5 (31%)
Furosemide	14 (88%)

*Only 6 patients underwent right heart catheterization as part of this study protocol.

SD: standard deviation; F: female; M: male; PAH: pulmonary arterial hypertension; RAP: right atrial pressure; mPAP: mean pulmonary arterial pressure; PCWP: pulmonary capillary wedge pressure; CI: cardiac index; PVR: pulmonary vascular resistance; 6MWD: six-minute walked distance; WHO: world health organization.

### Quality of life and ease of administration assessment

**Fig 2:**
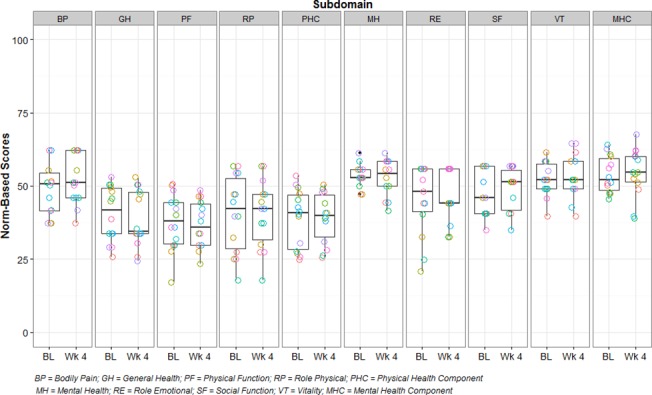
Individual SF-36 domain scores at baseline and after 4 weeks of treatment with the reformulated product. The box plots are schematic box and whisker plots such that the whisker above represents the largest observed value that falls within for the upper fence (3^rd^ quartile + 1.5 * IQR) and the whisker below represents the smallest observed value that falls within the lower fence (1^st^ quartile—1.5 * IQR). IQR = inner quartile range calculated as 3^rd^ quartile value minus 1^st^ quartile value. PF: physical functioning; RP: role physical; RE: role emotional; VT: vitality; MH: mental health; SF: social functioning; BP: bodily pain; GH: general health; PHC: mean physical health component score; MHC: mean mental health component score.

The mean physical health component score of the SF-36 survey remained unchanged (−0.1 (4.3) from baseline (38.0 (9.7)) to Week 4 (37.9 (8.6)). Conversely, the mean mental health component score slightly increased (+1.1 (6.5)) from baseline (54.4 (6.3)) to Week 4 (55.5 (8.1)). Specific domain and component scores are presented in **Fig. 2**.

Ease of administration and changes in HRQoL, in particular activities of daily living assessment, were also assessed using a 15-question study-specific questionnaire (**S1 QoLassessment**). There were small improvements in the mean scores for the majority of the study-specific questionnaire items. Fourteen of the sixteen subjects (88%) preferred the reformulated product to the currently marketed Flolan. There was no significant change in the dose of reformulated product from baseline (mean dose of 36.1 (10.1) ng/kg/min) to the time of discharge (mean dose of 36.3 (16.5) ng/kg/min). Only 1 subject had his dose slightly reduced by 1 ng/kg/min at baseline prior to discharge due to nausea. The dose of reformulated product also remained stable after 4 weeks of treatment (mean dose of 36.8 (16.9) ng/kg/min).

### Secondary and exploratory endpoints

**Fig 3:**
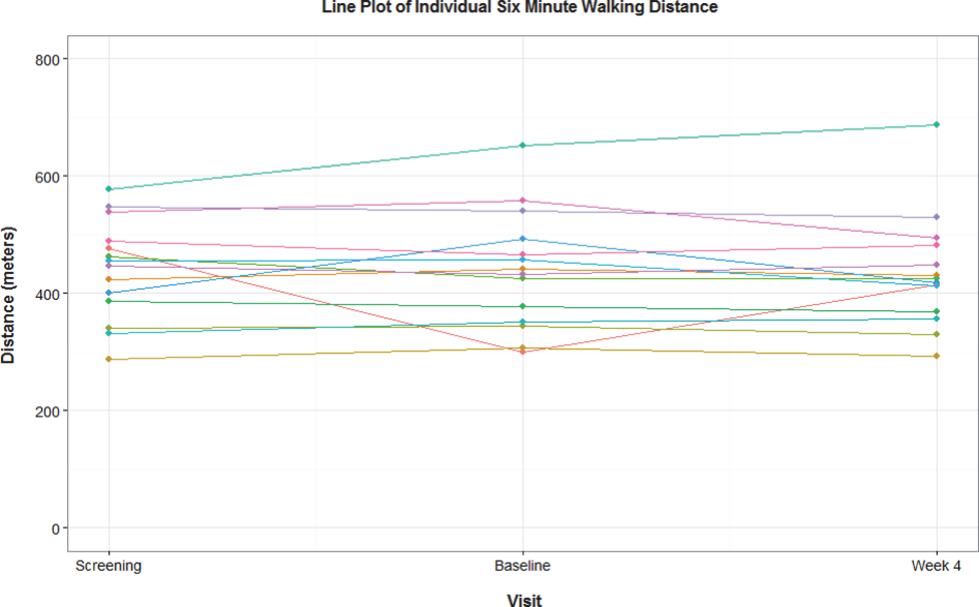
Individual 6 Minute Walk distance at the time of screening, at baseline and after 4 weeks of treatment with the reformulated product.

**Fig 4:**
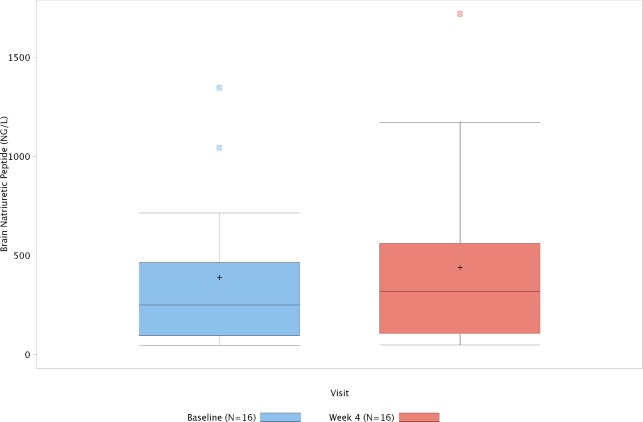
Mean N-terminal-pro B-type natriuretic peptide levels at baseline and after 4 weeks of treatment with the reformulated product. The box plots are schematic box and whisker plots such that the whisker above represents the largest observed value that falls within for the upper fence (3^rd^ quartile + 1.5 * IQR) and the whisker below represents the smallest observed value that falls within the lower fence (1^st^ quartile—1.5 * IQR). IQR = inner quartile range calculated as 3^rd^ quartile value minus 1^st^ quartile value.

After 4 weeks of treatment, the 6MWD remained unchanged compared to baseline (439 (100) versus 440 (85) meters). One subject (6%) had a >10% increase and 2 subjects (13%) had a >10% decrease in 6MWD from baseline (**Fig. 3**). A slight decrease in mean Borg dyspnoea index was seen from screening to baseline (−0.46 (1.12)), while a slight increase was seen from baseline to week 4 (+0.36 (0.53)), which resulted in a similar mean BDI at screening (3.04 (2.28)) compared with Week 4 (2.93 (2.08)). An improvement in WHO functional class was reported for 1 subject (6%) from baseline to week 4. No subjects deteriorated during the course of the treatment period. Mean NT-Pro BNP was lower at week 4 compared with baseline (606 (800)ng/L) versus 705 (1315) ng/L) (**Fig. 4**). Six subjects were enrolled at centres where haemodynamic measurements at baseline and 1 to 2 hours post-transition to newly formulated Flolan was considered as standard of care practice and concurred with the local guidelines. No changes in mPAP (from 47 (19) to 44 (15) mmHg), CI (from 2.9 (0.8) to 2.9 (0.8) L/min/m^2^) and PVR (from 8.4 (5.5) to 6.9 (3.6) mmHg/L/min) were seen from baseline to 2 hours post transition.

### Safety

The number and percentage of subjects with any adverse event were higher during the treatment period (9 subjects) compared with the run-in period (3 subjects). The majority of adverse events were mild or moderate in intensity. The only severe adverse events reported were device-related infections (2 subjects during the run-in period) and back pain (1 subject during the treatment period). The number and percentage of subjects with adverse events considered related to study drug by the investigator (1 subject and 3 subjects in the run-in and treatment periods, respectively) or who had serious adverse events (2 subjects and 1 subject, respectively) were low and similar in the 2 study periods. None of the serious adverse events were considered related to study drug by the investigator, and none of the 16 subjects in the study had any clinical chemistry analyse values outside the clinical concern reference range at any point during the study. No changes in systolic and diastolic blood pressure and heart rate were observed from baseline to week 4.

## Discussion

Transition from the currently marketed Flolan to the new reformulated Flolan is safe but not associated with a clinically meaningful difference in HRQoL, as measured by the SF-36. However, subjects reported improvements in some activities of daily living and ease of administration with the reformulated product, and overall preference for the reformulated product. Moreover, the efficacy (as measured by 6MWD, BDI, WHO functional class, NT-proBNP and haemodynamic parameters in the few subjects where the procedure was performed) and the safety profile of the reformulated product were similar to the currently marketed Flolan.

Intravenous epoprostenol (Flolan, Glaxo-SmithKline) became the first approved treatment for advanced PAH in the mid-1990’s. Epoprostenol has antithrombotic properties and is a potent vasodilator of both the systemic and pulmonary arteries [11]. The first randomized clinical trial in PAH showed that epoprostenol improved quality of life, hemodynamics, exercise tolerance, and survival over a 12-week period [6]. Observational studies also documented the long-term effects of epoprostenol on survival in PAH [12–13]. Initially proposed as a bridge to lung transplantation, epoprostenol has become the standard of care treatment for patients with advanced PAH [14]. However, safe and effective administration of Flolan by continuous intravenous infusion is complex and requires considerable commitment from patients. Currently marketed Flolan requires reconstitution and dilution every 2 days and the reconstituted solution may only be administered up to 24 hours when it is maintained between a temperature of 2°C and 8°C during infusion. Therefore, the use of a cold pack that is changed at least every 12 hours is required.

The present study assessed the effects of a transition from currently marketed epoprostenol to reformulated product on HRQoL and ease of administration with the presumption that less frequent Flolan reconstitution and the elimination of cold pack requirement would improve patients’ HRQoL and ease of administration. The SF-36 was used on a descriptive basis to assess whether the change in Flolan formulation would impact HRQoL. The magnitude and pattern of directional change in the group mean domain scores did not indicate any significant impact on HRQoL between the reformulated product and the currently marketed Flolan. For the study-specific questionnaire, however, subjects reported improvements in aspects of daily living and ease of administration of medication with the reformulated product compared to the currently marketed Flolan. Plus, most stated that they preferred the reformulated product. Surprisingly, most subjects in this study continued to reconstitute their Flolan daily although the reformulated product allowed the flexibility to only reconstitute every sixth day; this may have been physician directed.

Clinical dose adjustment for deterioration with transition did not occur. Dose did not change from the first dose of reformulated product at baseline to within 48 hours after the first dose with the reformulated product. The minimal differences in efficacy parameters (as measured by 6MWD, BDI and WHO functional class) before and after the transition to the reformulated product, were not considered to be clinically meaningful. Additionally, in the few subjects where the procedure was performed, haemodynamic parameters were similar before and after the switch. Taken together, these data suggest that the increase in pH of the reformulated product had no adverse effects on the potency of the product. This was as expected as there has been no change made to the vial that contains the lyophilised epoprostenol, the active ingredient in Flolan.

Similar to previous trials with eoprostenol, adverse events related to the drugs known side effects occurred before and after transition. The majority of adverse events were mild or moderate in intensity and none of them led to treatment withdrawal. The increase in the pH of the diluent did not increase the frequency or severity of injection site reactions nor catheter-related sepsis. It is noteworthy, however, that the open-label design and the short duration of this study do not allow drawing clear conclusions about the safety and efficacy of the reformulated Flolan.

## Conclusion

In conclusions, the present study documented that the reformulated product was not associated with significant improvement in HRQoL compared with the currently marketed Flolan as measured by the SF-36. However, most subjects preferred the reformulated product to the currently marketed Flolan. Moreover, the 2 formulations of Flolan had short-term similar safety and efficacy profiles.

## Supporting Information

S1 Protocol Trial Protocol. (PDF)

S1 CONSORT Checklist CONSORT Checklist. (PDF)

S1 QoLassessment A study-specific questionnaire evaluating ease of administration. (DOCX)
