# Supplementary material for: Quality of Life, Safety and Efficacy Profile of Thermostable Flolan in Pulmonary Arterial Hypertension
Source: PLoS One. 2015 Mar 20;10(3):e0120657. doi: 10.1371/journal.pone.0120657 (PMC4368561; doi:10.1371/journal.pone.0120657)
Supplement: S1 Protocol — (PDF) [file pone.0120657.s001.pdf]

Division: Worldwide Development
Retention Category: GRS019
Information Type: Protocol Amendment

**Title:**	A Single-arm, Open Label Study Evaluating the Impact on Life-style of a New Thermo Stable Formulation of FLOLAN™ in Subjects with Pulmonary Arterial Hypertension (PAH): Protocol Amendment 1

Compound Number: AH21461 (4AU76)

Effective Date: 19-JAN-2012

Protocol Amendment Number: 01

Description:

This document describes the original protocol with the changes resulting from protocol amendment 1 included.

FLOLAN (epoprostenol sodium) is an effective treatment for pulmonary arterial hypertension (PAH); however, administration of FLOLAN is complex and requires a considerable level of commitment from patients. As a result, a new formulation of FLOLAN diluent has been developed, which is more stable at ambient temperatures and allows less frequent reconstitution and dilution compare to current FLOLAN formulation.

This will be a multicenter, open label, single-arm study in patients who are already receiving FLOLAN for the treatment of PAH. The study will include a screening visit, a 4-week run-in period with existing FLOLAN treatment, a 4-week treatment period with the new reformulated FLOLAN diluent product and a follow-up visit. An optional extension phase of the study will also be available to subjects.

Subject: Flolan, Pulmonary Arterial Hypertension, New Thermo Stable Formulation, Quality of Life

Author: Cirkel, Deborah T (MPC CPSE); Shaddinger, Bonnie C (MPC CPSE); Paruchuru, Pratap K (MPC); Froloshki, Borislav S (MPC BDS); Vettese-Dadey, Michelle MPC CPSE); Plastino, Janet E MPC Clinical Data Management); Spezzi, Andrea (Rare Disease Unit)

Copyright 2012 the GlaxoSmithKline group of companies. All rights reserved. Unauthorised copying or use of this information is prohibited.

Revision Chronology:

2011N113389/00	2011-MAY-25	Original
2011N113389/01	2012-JAN-19	Amendment No.: 01. Correction of some minor typographical errors.

**Sponsor Signatory:**	**Signature:**	**Date:**
Andrea Spezzi, MD, MFPM<br>VP and Medicines Development Leader, Rare Disease Unit	[signature]	19/01/12

SPONSOR INFORMATION PAGE

Clinical Study Identifier: FLR115332

GlaxoSmithKline
Iron Bridge Road
Stockley Park West, Uxbridge, Middlesex, UB11 1BU, UK
Telephone: +44 (0)208 990 9000

Sponsor Medical Monitor Contact Information and Sponsor Serious Adverse Events (SAE) Contact Information:

Pratap K. Paruchuru, FRCS, MFPM
Clinical Development Physician
GlaxoSmithKline
Stockley Park

Regulatory Agency Identifying Number(s): IND 016459;
EudraCT Number 2011-002943-92

INVESTIGATOR AGREEMENT PAGE

For protocol number FLR115332 Protocol Amendment 01

I confirm agreement to conduct the study in compliance with the protocol, as amended by this protocol amendment.

I acknowledge that I am responsible for overall study conduct. I agree to personally conduct or supervise the described clinical study.

I agree to ensure that all associates, colleagues and employees assisting in the conduct of the study are informed about their obligations. Mechanisms are in place to ensure that site staff receives the appropriate information throughout the study.

Investigator Name: ____________________________

____ ____________________

Investigator Signature Date

TABLE OF CONTENTS

PAGE

LIST OF ABBREVIATIONS 8

PROTOCOL SUMMARY 10

1. INTRODUCTION 14
   1.1. Background 14
   1.2. Rationale 14

2. OBJECTIVE(S) 16
   2.1. Primary 16
      2.1.1. Secondary 16
      2.1.2. Exploratory 16

3. INVESTIGATIONAL PLAN 16
   3.1. Study Design 16
   3.2. Discussion of Design 17

4. SUBJECT SELECTION AND WITHDRAWAL CRITERIA 17
   4.1. Number of Subjects 17
   4.2. Inclusion Criteria 18
   4.3. Exclusion Criteria 18
   4.4. Withdrawal Criteria 19

5. STUDY TREATMENTS 20
   5.1. Investigational Product 20
   5.2. Treatment Assignment 20
   5.3. Blinding 21
   5.4. Product Accountability 21
   5.5. Treatment Compliance 21
   5.6. Concomitant Medications and Non-Drug Therapies 21
      5.6.1. Permitted Medications and Non-Drug Therapies 21
      5.6.2. Prohibited Medications and Non-Drug Therapies 21
   5.7. Treatment after the End of the Study 22
   5.8. Treatment of Study Treatment Overdose 22

6. STUDY ASSESSMENTS AND PROCEDURES 22
   6.1. Critical Baseline Assessments 26
   6.2. Efficacy 26
   6.3. Safety 26
      6.3.1. Physical examination 26
      6.3.2. Electrocardiogram 26
      6.3.3. Vital Signs 26
      6.3.4. Clinical Laboratory Tests 27
      6.3.5. Contraception Requirements 27
      6.3.6. Adverse Events 28
         6.3.6.1. Definition of an AE 28
         6.3.6.2. Definition of a SAE 29

6.3.7. Laboratory and Other Safety Assessment Abnormalities Reported as AEs and SAEs 30
6.3.8. Pregnancy 31
6.3.9. Time Period and Frequency of Detecting AEs and SAEs 31
6.3.10. Prompt Reporting of Serious Adverse Events and Other Events to GSK 31
6.3.10.1. Regulatory reporting requirements for SAEs 32
6.4. Pharmacogenetics 32

7. DATA MANAGEMENT 32

8. DATA ANALYSIS AND STATISTICAL CONSIDERATIONS 33
8.1. Hypotheses 33
8.2. Study Design Considerations 33
8.2.1. Sample Size Assumptions 33
8.2.2. Sample Size Sensitivity 33
8.2.3. Sample Size Re-estimation 33
8.3. Data Analysis Considerations 33
8.3.1. Analysis Populations 33
8.3.2. Analysis Data Sets 33
8.3.3. Treatment Comparisons 33
8.3.3.1. Primary Measurements of Interest 33
8.3.3.2. Secondary Measurements of Interest 34
8.3.3.3. Other Measurements of Interest 34
8.3.4. Interim Analysis 34
8.3.5. Key Elements of Analysis Plan 35
8.3.5.1. Quality of life analysis 35
8.3.5.2. Safety Analyses 35
8.3.5.3. Efficacy Analyses 35
8.3.5.4. Other Analyses 35
8.3.5.5. Pharmacogenetic Analyses 35

9. STUDY CONDUCT CONSIDERATIONS 36
9.1. Posting of Information on Clinicaltrials.gov 36
9.2. Regulatory and Ethical Considerations, Including the Informed Consent Process 36
9.3. Quality Control (Study Monitoring) 36
9.4. Quality Assurance 37
9.5. Study and Site Closure 37
9.6. Records Retention 37
9.7. Provision of Study Results to Investigators, Posting to the Clinical Trials Register and Publication 38
9.8. Independent Data Monitoring Committee (IDMC) 39

10. REFERENCES 40

11. APPENDICES 41
11.1. Appendix 1: PGx 41
11.2. Appendix 2: Protocol Changes 46

LIST OF ABBREVIATIONS

6MWD	6 minute walking distance test
ACE	Angiotensin-converting enzymes
AE	Adverse Event
ALT	alanine aminotransferase
AST	aspartate aminotransferase
BDI	Borg Dyspnoea Index
BUN	Blood urea nitrogen
cGMP	Cyclic guanosine monophosphate
CI	Cardiac index
CO2	Bicarbonate
CPK	Creatine phosphokinase
ECG	Electrocardiogram
eCRF	Electronic Case Report Form
ERA	Endothelin receptor antagonist
FDA	Food and Drug Administration
FC	Functional class
FSH	Follicle-stimulating hormone
GCP	Good Clinical Practice
GGT	Gamma glutamyl transferase
GSK	GlaxoSmithKline
IDMC	Independent Data Monitoring Committee
IEC	Independent Ethics Committee
IRB	Institutional Review Board
ITT	Intention-to-treat
IV	intravenous
LDH	Lactate dehydrogenase
LEVDP	Left ventricular end diastolic pressure
LFT	Liver function test
LH	Luteinizing hormone
MCH	mean corpuscular haemoglobin
MCHC	mean corpuscular haemoglobin concentration
MCV	mean corpuscular volume
MedDRA	Medical Dictionary for Regulatory Activities
mPAP	mean pulmonary arterial pressure
MSDS	Material Safety Data Sheet
NT-Pro BNP	N-Terminal pro-B-type Natriuretic Peptide
PAH	Pulmonary Arterial Hypertension
PCWP	Pulmonary capillary wedge pressure
PDE-5	Phosphodiesterase type 5
PGx	Pharmacogenetic
PVR	Pulmonary vascular resistance
QOL	Quality of Life
RAP	Right atrial pressure
RHC	Right heart catheterisation

RVF	Right ventricular failure
SAE	Serious Adverse Event
SaO2	Oxygen saturation
SF-36	Short Form-36
SPM	Study Procedures Manual
ULN	Upper limit of normal
WHO	World Health Organization

Trademark Information

Trademarks of the GlaxoSmithKline group of companies
FLOLAN

Trademarks not owned by the GlaxoSmithKline group of companies
None

PROTOCOL SUMMARY

Rationale

FLOLAN™ (epoprostenol sodium) for injection is a sterile sodium salt formulated for intravenous (IV) administration. Epoprostenol (also called PGI_2, PGX, prostacyclin), a metabolite of arachidonic acid, is a naturally occurring prostaglandin with potent vasodilatory activity and inhibitory activity of platelet aggregation. Epoprostenol has two major pharmacological actions 1) direct vasodilatation of pulmonary and systemic arterial vascular beds, and 2) inhibition of platelet aggregation.

FLOLAN is an effective treatment for pulmonary arterial hypertension (PAH), however, safe and effective administration is complex and requires a considerable level of commitment from patients. The current marketed formulation of FLOLAN requires reconstitution and dilution every two days and the reconstituted solution may only be administered up to 24 hours when it is maintained between a temperature of 2° and 8°C (36° to 46° F) during infusion, thereby, necessitating the use of a cold pack. In addition, the cold pack used to maintain the temperature of the reconstituted solution must be changed every 12 hours.

In 1993, Burroughs Wellcome Co (BW), one of the legacy companies of GlaxoSmithKline (GSK) and the original developer of FLOLAN, attempted to reformulate the freeze dried epoprostenol sodium so that it could be reconstituted using sterile water for injection rather than with the specially formulated diluent. This formulation change included substantial changes to the concentrations of mannitol, glycine, sodium chloride, and sodium hydroxide in the final, reconstituted injection. Changes were made to the vial that contained the active drug substance, which resulted in the patient receiving more mannitol and sodium hydroxide and less glycine and sodium chloride daily with the single vial formulation compared to the original formulation, therefore, the further work on this failed formulation was terminated a year later.

GSK is currently developing a new formulation of FLOLAN diluent, which is more stable at ambient temperatures as the pH of the diluent has been increased from 10.5 to 12. No change will be made to the vial that contains the lyophilised epoprostenol. The only change will be the pH of the diluent. The new formulation may be reconstituted and diluted every 6 days and is stable for 24 hours up to 35°C (95° F) and for 48 hours up to 25°C (77° F); it does not, therefore, require the use of a cold pack or frequent changes of the cassette. In this way, it is anticipated that the new formulation will provide an added level of convenience to patients through reduction in the frequency of reconstitution/dilution, and elimination of the need for a cold pack, even in countries with high ambient temperatures. The change has been limited only to the diluent with the active part of the formulation remaining intact. Therefore, it is not expected to have any impact on the pharmacodynamic actions of FLOLAN, and the clinical profile is expected to be the same as that of the current formulation. It is not anticipated that any dose titration or maintenance dose adjustments will be required in stable patients who are transitioning from the old formulation, however, it is not infrequent due to the fragility of this patient population that in certain circumstances such as disease progression, changes might need to be implemented. However, it is not expected that immediate changes would be required due to the switch of the formulation.

The purpose of this study is to demonstrate that the new formulation of FLOLAN will result in greater convenience and ease of administration thereby improving quality of life for those patients transitioning from the old formulation to the new thermo stable formulation. The study will also collect dose requirements, safety, tolerability and efficacy data as secondary objectives.

Objective(s)

Primary

- To describe the effect of the new thermo stable formulation of FLOLAN on quality of life in patients switching from the currently marketed FLOLAN to the new thermo stable formulation
- To determine the dose titration requirement in patients switching from the currently marketed FLOLAN to the new thermo stable formulation

Secondary

- To evaluate the safety and tolerability of the thermo stable formulation of FLOLAN
- To evaluate the efficacy of thermo stable formulation of FLOLAN

Exploratory

- To evaluate the effect of the new thermo stable formulation of FLOLAN on hemodynamic parameters in a subset of subjects

Study Design

This will be a multicenter, open label, single-arm study in subjects who are currently receiving FLOLAN for the treatment of PAH.

Approximately twenty subjects will be enrolled in the study in order to have at least 15 subjects to complete the study. Given the nature of the disease, the sample size for this study was chosen based on feasibility and no specific hypothesis testing is planned.

The study will include a screening visit, a 4-week run-in period with existing FLOLAN treatment, a 4-week treatment period with the new reformulated FLOLAN diluent product and an optional extension period. After the initial 4 week run-in period, subjects will be admitted to the clinic for baseline assessments and for switching to study medication. Subjects will remain in hospital for a minimum of 6 hours to ensure clinical and hemodynamic stability prior to discharge. Subjects may stay in hospital for up to 24-48 hours after switching to the new reformulated FLOLAN diluent at the discretion of the investigator. Dose titration requirement will be assessed at the time of discharge. Planned admission to a hospital setting to facilitate the transition of formulations will only be done per local guidelines or medical practice. Hemodynamic parameters will be obtained in a subgroup of subjects enrolled in centres where the collection of

hemodynamic data is considered part of the standard of care. Subjects who completed the 4-week treatment period will have the option to continue study treatment in an extension phase of the study. For subjects who are participating in the extension, the follow-up visit will occur at the end of the extension period.

The regimens to be studied are:

Regimen	Period	Description
A	run-in	Current marketed FLOLAN (epoprostenol sodium+ pH 10.5 diluent) for Injection
B	treatment	New reformulated FLOLAN diluent (epoprostenol sodium + pH 12 diluent) for Injection
B	extension (optional)	New reformulated FLOLAN diluent (epoprostenol sodium + pH 12 diluent) for Injection

The total study duration including screening, run-in, treatment and follow-up will be approximately 9 to 11 weeks in the main portion of study excluding the extension phase.

Study Endpoints/Assessments

As this is a descriptive study with no formal statistical testing planned, study endpoints will be summarized in tabular and graphical format. Individual patient listings will be provided.

Primary

- Quality of life assessment using SF-36 questionnaire
- Ease of administration and changes in quality of life, in particular activities of daily living assessment using study specific questionnaire
- Change from baseline in the dose of thermo stable FLOLAN formulation at the time of discharge at a minimum of 6 hours or per local guidelines/practices

Secondary

Safety and tolerability will be assessed based on a review of the following parameters:

- Adverse events
- Serious Adverse Events
- Vital signs: systolic and diastolic blood pressure, heart rate
- Clinical laboratory tests (clinical chemistry, hematology, and urinalysis)
- Infusion site reactions including erythema, excoriation, induration, skin necrosis or signs of local sepsis

Efficacy will be assessed based on a review of the following parameters:

- Six minute walk distance test (6MWD) after 4-weeks of treatment
- Breathlessness after 6MWD – Borg Dyspnoea Index (BDI)
- World Health Organization [WHO] functional class at baseline and after 4-weeks of treatment

Exploratory

- Hemodynamic parameters (Mean Pulmonary Artery Blood Pressure in mmHg [mPAP], Pulmonary Vascular resistance [PVR], Cardiac Index [CI], Right Atrial Pressure [RAP]) at baseline, and at 1 to 2 hours in a subset of the population in centres where the procedure is normal practise and concurs with local guidelines.

1. INTRODUCTION

1.1. Background

Pulmonary arterial hypertension (PAH) is a chronic debilitating disease characterised by a progressive increase of pulmonary vascular resistance (PVR) leading to right ventricular failure (RVF) and premature death. PAH carries an extremely poor prognosis in untreated patients with a median survival of approximately 2 years. The survival is much worse in patients with PAH associated with connective tissue disease with the median survival of approximately 12 months.

The goals of therapy include improvement of symptoms, quality of life and prevention of progression of the disease. Effective treatments that target the pathophysiology exist including prostanoids, phosphodiesterase type 5 (PDE5) inhibitors and endothelin receptor antagonists, of which the vasodilator prostanoids are the longest established. These target potential imbalances in the prostaglandin signalling pathway in PAH, stimulate vasodilatation and anti-proliferative effects [Jones, 1995; Humbert, 2004].

The anti-platelet effect of prostanoids ameliorates the microthrombotic consequences that are a feature of PAH. Of the prostanoids, most evidence exists for epoprostenol. Based on published literature, this is the only therapy for PAH that has been clearly demonstrated to reduce mortality as well as reduce symptoms of the disease [McLaughlin, 2002; Sitbon, 2002]. It is recommended as first line targeted therapy in patients with the most severe (WHO class IV) symptoms and as an option in those with class III symptoms [Galie, 2004]. In clinical practice the majority of use is restricted to patients with functional class IV symptoms owing to the requirement that the drug be delivered by continuous intravenous infusion.

Alternative prostanoids, such as subcutaneous trepostinil are available, and there is evidence of clinical benefit [Simonneau, 2002]. Although subcutaneous administration avoids some of the infectious complications of intravenous administration, local tolerability (erythema, induration, and rash) is a significant issue for many patients (~35%) [Horn, 2002]. Inhaled iloprost has to be administered 6-9 times a day making it less suitable for many patients. Thus, the prostanoid class has demonstrable efficacy, but its pharmacokinetic characteristics make delivery challenging. This has led to the search for alternative formulations of Epoprostenol which can be delivered more simply (eg: without the need for frequent changes of the cassette, the need for ice packs and longer stability at room temperatures) which would provide convenience and enhance the quality of life for these patients.

1.2. Rationale

FLOLAN (epoprostenol sodium) for injection is a sterile sodium salt formulated for intravenous (IV) administration. Epoprostenol (also called PGI_2, PGX, prostacyclin), a metabolite of arachidonic acid, is a naturally occurring prostaglandin with potent vasodilatory activity and inhibitory activity of platelet aggregation. Epoprostenol has two major pharmacological actions 1) direct vasodilatation of pulmonary and systemic arterial vascular beds, and 2) inhibition of platelet aggregation.

FLOLAN is an effective treatment for pulmonary arterial hypertension (PAH), however, safe and effective administration is complex and requires a considerable level of commitment from patients. The current marketed formulation of FLOLAN requires reconstitution and dilution every two days and the reconstituted solution may only be administered up to 24 hours when it is maintained between a temperature of 2° and 8°C (36° to 46° F) during infusion, thereby, necessitating the use of a cold pack. In addition, the cold pack used to maintain the temperature of the reconstituted solution must be changed every 12 hours.

In 1993, Burroughs Wellcome Co (BW), one of the legacy companies of GlaxoSmithKline (GSK) and the original developer of FLOLAN, attempted to reformulate the freeze dried epoprostenol sodium so that it could be reconstituted using sterile water for injection rather than with the specially formulated diluent. This formulation change included substantial changes to the concentrations of mannitol, glycine, sodium chloride, and sodium hydroxide in the final, reconstituted injection. Changes were made to the vial that contained the active drug substance, which resulted in the patient receiving more mannitol and sodium hydroxide and less glycine and sodium chloride daily with the single vial formulation compared to the original formulation, therefore, the further work on this failed formulation was terminated a year later.

GSK is currently developing a new formulation of FLOLAN diluent which is more stable at ambient temperatures as the pH of the diluent has been increased from 10.5 to 12. No change will be made to the vial that contains the lyophilised epoprostenol. The only change will be the pH of the diluent. The new formulation may be reconstituted and diluted every 6 days and is stable for 24 hours up to 35°C (95° F) and for 48 hours up to 25°C (77° F); it does not, therefore, require the use of a cold pack or frequent changes of the cassette. In this way, it is anticipated that the new formulation will provide an added level of convenience to patients through reduction in the frequency of reconstitution/dilution, and elimination of the need for a cold pack, even in countries with high ambient temperatures. The change has been limited only to the diluent with the active part of the formulation remaining intact. Therefore, it is not expected to have any impact on the pharmacodynamic actions of FLOLAN, and the clinical profile is expected to be the same as that of the current formulation. It is not anticipated that any dose titration or maintenance dose adjustments will be required in stable patients who are transitioning from the old formulation, however, it is not infrequent due to the fragility of this patient population that in certain circumstances such as disease progression, changes might need to be implemented. However, it is not expected that immediate changes would be required due to the switch of the formulation.

The purpose of this study is to demonstrate that the new formulation of FLOLAN will result in greater convenience and ease of administration thereby improving quality of life for those patients transitioning from the old formulation to the new thermo stable formulation. The study will also collect dose requirements, safety, tolerability and efficacy data as secondary objectives.

2. OBJECTIVE(S)

2.1. Primary

- To describe the effect of the new thermo stable formulation of FLOLAN on quality of life in patients switching from the currently marketed FLOLAN to the new thermo stable formulation
- To determine the dose titration requirement in patients switching from the currently marketed FLOLAN to the new thermo stable formulation

2.1.1. Secondary

- To evaluate the safety and tolerability of the thermo stable formulation of FLOLAN
- To evaluate the efficacy of thermo stable formulation of FLOLAN

2.1.2. Exploratory

- To evaluate the effect of the new thermo stable formulation of FLOLAN on hemodynamic parameters in a subset of subjects

3. INVESTIGATIONAL PLAN

3.1. Study Design

Protocol waivers or exemptions are not allowed. Therefore, adherence to the study design requirements, including those specified in the Time and Events Table, are essential.

This will be a multicenter, open label, single-arm study in subjects who are currently receiving FLOLAN for the treatment of PAH.

Approximately twenty subjects will be enrolled in the study in order to have at least 15 subjects to complete the study. Given the nature of the disease the sample size for this study was chosen based on feasibility and no specific hypothesis testing is planned.

The study will include a screening visit, a 4-week run-in period with existing FLOLAN treatment, a 4-week treatment period with the new reformulated FLOLAN diluent product and an optional extension period. After the initial 4 week run-in period, subjects will be admitted to the clinic for baseline assessments and for switching to study medication. Subjects will remain in hospital for a minimum of 6 hours to ensure clinical and hemodynamic stability prior to discharge. Subjects may stay in hospital for up to 24-48 hours after switching to the new reformulated FLOLAN diluent at the discretion of the investigator. Dose titration requirement will be assessed at the time of discharge. Planned admission to a hospital setting to facilitate the transition of formulations will only be done per local guidelines or medical practice. Hemodynamic parameters will be obtained in a subgroup of subjects enrolled in centres where the collection of

hemodynamic data is considered part of the standard of care. Subjects who completed the 4-week treatment period will have the option to continue study treatment in an extension phase of the study. For subjects who are participating in the extension, the follow-up visit will occur at the end of the extension period.

The regimens to be studied are:

Regimen	Period	Description
A	run-in	Current marketed FLOLAN (epoprostenol sodium+ pH 10.5 diluent) for Injection
B	treatment	New reformulated FLOLAN diluent (epoprostenol sodium + pH 12 diluent) for Injection
B	extension (optional)	New reformulated FLOLAN diluent (epoprostenol sodium + pH 12 diluent) for Injection

The total study duration including screening, run-in, treatment and follow-up will be approximately 9 to 11 weeks in the main portion of study excluding the extension phase.

Supplementary study conduct information not mandated to be present in this protocol is provided in the accompanying Study Procedures Manual (SPM). The SPM will provide the site personnel with administrative and detailed technical information that does not impact subject safety.

3.2. Discussion of Design

PAH is a life threatening disease and FLOLAN is an important treatment for many patients, including those with the most severe disease. The purpose of this study is to evaluate the advantages of the new thermo stable FLOLAN formulation in terms of ease of administration, for example, decreasing the frequency of reconstitutions, eliminating the need to carry ice packs, and thereby improving quality of life for patients. Study duration of 4 weeks was chosen to allow patients sufficient length of time to learn to set up and administer the new formulated FLOLAN while allowing sufficient time to appreciate any meaningful differences the new formulation may bring to their activities of daily living. In addition the secondary endpoints of 6MWD and BDI are similar to previous PAH studies with other agents. As the study also seeks to support that the new formulation of FLOLAN diluent is functionally comparable to the existing formulation, it is important that subjects enrolled are sufficiently stable to allow meaningful comparison between baseline and after treatment. The study will not be powered and descriptive statistics will be used.

4. SUBJECT SELECTION AND WITHDRAWAL CRITERIA

4.1. Number of Subjects

Approximately twenty subjects will be enrolled in the study in order to have at least 15 subjects to complete the study. Given the nature of the disease, the sample size for this study was chosen based on feasibility and no specific hypothesis testing is planned.

4.2. Inclusion Criteria

Specific information regarding warnings, precautions, contraindications, adverse events, and other pertinent information on the GSK investigational product or other study treatment that may impact subject eligibility is provided in the product label.

Deviations from inclusion criteria are not allowed because they can potentially jeopardize the scientific integrity of the study, regulatory acceptability or subject safety. Therefore, adherence to the criteria as specified in the protocol is essential.

Subjects eligible for enrolment in the study must meet all of the following criteria:

1. Adult male or female at least 18 to 75 years at the time of screening.
2. Subjects must have been on FLOLAN therapy for pulmonary arterial hypertension (PAH) as approved in the product label.
3. Subjects must be on stable doses of their existing FLOLAN treatment for a minimum of 3 months prior to screening;
4. Subjects must be on stable doses of any current PAH treatments other than FLOLAN in the last 30 days;
5. Subjects must walk a distance of at least 150 meters during six-minute walk distance test (6MWD). This test must be completed during the Screening Visit;
6. A female subject is eligible to participate if she is of:
   - Child-bearing potential must have a negative urine pregnancy at screening and baseline and agrees to use one of the contraception methods listed in Section 6.3.5 for an appropriate period of time (as determined by the product label or investigator) prior to the start of dosing to sufficiently minimize the risk of pregnancy at that point. Female subjects must agree to use contraception until the end of follow-up visit.
   - Non-childbearing potential defined as pre-menopausal females with a documented tubal ligation or hysterectomy; or postmenopausal defined as 12 months of spontaneous amenorrhea [in questionable cases a blood sample with simultaneous follicle stimulating hormone (FSH) > 40 MlU/ml and estradiol < 40 pg/ml (<147 pmol/L) is confirmatory].
7. Subjects must be competent to understand the information given in the Institutional Review Board (IRB) or Independent Ethics Committee (IEC) approved informed consent form and must sign the form prior to the initiation of any study procedures.

4.3. Exclusion Criteria

Deviations from exclusion criteria are not allowed because they can potentially jeopardize the scientific integrity of the study, regulatory acceptability or subject safety. Therefore, adherence to the criteria as specified in the protocol is essential.

Subjects meeting any of the following criteria must not be enrolled in the study:

1. Subjects who are given FLOLAN for a condition or in a manner that is outside the approved indication.
2. Subjects with congestive heart failure arising from severe left ventricular dysfunction.
3. Subjects, with or without supplemental oxygen, who have a resting arterial oxygen saturation (SaO2) <90% as measured by pulse oximetry at screening.
4. Subjects have been hospitalized as an emergency or visited the emergency room for a condition related to PAH or treatment for PAH in the last 3 months.
5. The subject's clinical condition is such that they are not expected to remain clinically stable for the duration of the study.
6. Female subjects who are pregnant or breastfeeding.
7. Subjects who have demonstrated noncompliance with previous medical regimens.
8. Subjects who have a history of abusing alcohol or illicit drugs within 1 year.
9. Subjects with a diagnosis of active hepatitis (hepatitis B surface antibody and hepatitis C antibody).
10. Subjects who have participated in a clinical study involving another investigational drug or device within four weeks before screening.
11. Subjects who had history malignancies within the past 5 years, with the exception of basal cell carcinoma of the skin or in situ carcinoma of the cervix.
12. Any concurrent condition that would affect the safety of the subject or in the opinion of the investigator it is not in the best interest of the patient to participate in the study.

4.4. Withdrawal Criteria

Subjects who do not tolerate treatment will be withdrawn from the study. Treatment for subjects withdrawn from the study will be implemented at the Investigator's discretion.

A subject may also be discontinued prior to completion of the study for the following reasons, but not limited to:

- Adverse event (AE) or serious adverse event (SAE) which in the opinion of the investigator requires withdrawal or is thought to be not in the best interests of the subject to continue participation
- Positive pregnancy test
- Consent withdrawn
- Lost to follow-up
- Non compliance to protocol procedures (protocol deviation or violation)
- Termination of study by Sponsor
- Investigator's discretion (The reason will be documented in eCRF.)

5. STUDY TREATMENTS

5.1. Investigational Product

The contents of the label will be in accordance with all applicable regulatory requirements.

GSK will not provide FLOLAN products during the run-in period.

GSK will provide the following vials containing:

- epoprostenol 0.5mg lyophile
- epoprostenol 1.5mg lyophile
- sterile glycine diluent (pH 12)

To prepare the new thermo stable product, patients will reconstitute and dilute one or more vials of epoprostenol lyophile (according to therapeutic need) with two vials of sterile glycine diluent (pH 12), giving 100mL of medication for each day of treatment. This reconstituted infusion is filled into medication cassettes prior to either refrigerated storage or immediate continuous intravenous infusion via a central venous catheter using an ambulatory infusion pump.

Above is a summary of the preparation and descriptions of the investigational product. Specific detailed instructions on dosage, administration, preparation and storage requirements will be provided in the SPM. FLOLAN product label will also be provided.

Under normal conditions of handling and administration, investigational product is not expected to pose significant safety risks to site staff. A Material Safety Data Sheet (MSDS) describing the occupational hazards and recommended handling precautions will be provided to site staff if required by local laws or will otherwise be available from GSK upon request.

Adequate precautions must be taken to avoid direct contact with the investigational product. The occupational hazards and recommended handling procedures are provided in the Material Safety Data Sheet (MSDS).

Investigational product must be stored in a secure area under the appropriate physical conditions for the product. Access to and administration of the investigational product will be limited to the investigator and authorized site staff. Investigational product must be dispensed or administered only to subjects enrolled in the study and in accordance with the protocol.

5.2. Treatment Assignment

All Subjects will be receiving the new thermo stable formulation of FLOLAN during the treatment phase.

5.3. Blinding

This will be a single-arm, open-label study and there will be no blinding.

5.4. Product Accountability

In accordance with local regulatory requirements, the investigator, designated site staff, or head of the medical institution (where applicable) must document the amount of investigational product dispensed and/or administered to study subjects, the amount returned by study subjects, and the amount received from and returned to GSK, when applicable. Product accountability records must be maintained throughout the course of the study.

5.5. Treatment Compliance

Compliance will be assessed by the investigator or designee and recorded in the eCRF.

5.6. Concomitant Medications and Non-Drug Therapies

All concomitant medications taken during the study will be recorded in the CRF with indication, dose information, and dates of administration.

5.6.1. Permitted Medications and Non-Drug Therapies

Subjects should benefit from the standard of care treatment in PAH, according to local standards and subject conditions. Standard medical treatment administered prior to screening may be maintained throughout the study.

Subjects taking PAH specific medications (see examples below) must have stable PAH therapy for at least 1 month prior to screening and the therapy will need to be maintained throughout the duration of treatment period. No changes to PAH-specific medication are allowed during the treatment period.

Examples of permitted PAH-specific medications, but not limited to:

- vasodilators such as angiotensin-converting enzymes (ACE) inhibitors, calcium channel blockers and nitric oxide
- cyclic guanosine monophosphate (cGMP)-specific phosphodiesterase type 5 (PDE-5) inhibitors such as sildenafil and tadalafil
- Endothelin receptor antagonist (ERA) such as ambrisentan and bosentan

5.6.2. Prohibited Medications and Non-Drug Therapies

During the study, no other prostanoid therapy or its reformulation will be permitted.

5.7. Treatment after the End of the Study

The investigator is responsible for ensuring that consideration has been given to the post-study care of the patient's medical condition whether or not GSK is providing specific post study treatment. The end of the study could be either after the 4-week treatment period or if the subject decided to participate in the optional extension phase, the end of extension phase then will mark the end of the study.

5.8. Treatment of Study Treatment Overdose

Please refer to the approved product label.

6. STUDY ASSESSMENTS AND PROCEDURES

Table 1 Time and Events Table

		4-week Run-in period	Treatment Phase						Extension Phase		
	Visit 1	Phone call (occurring 14 days prior to baseline)	Visit 2	Phone call	Un-scheduled Visit	Early withdraw	Visit 3	Follow-up Visit[1]	Phone call[2] at Month 3 (plus every 6 months)	Clinic Visit[3] at Month 6 (plus every 6 month)	End of Extension Study Visit or Un-scheduled Visit or Early withdraw
**Procedures**	**Screen[4]**	**Day-14**	**Baseline[5] (±7 d)**	**Day 3, 5, 7 & Week 2**			**Week 4 (±7 d)**		**Month 3 (±14 d)**	**Month 6 (±14 d)**	
Written Informed Consent	x										
Subject Demography	x										
Medical/Disease/Therapy History	x										
Inclusion/Exclusion Criteria	x										
**Efficacy Assessments**											
6MWD Test	x		x			x	x				
Borg Dyspnoea Index	x		x			x	x				
WHO Functional Class	x		x		x	x	x	x		x	x
QOL and study Assessment			x			x	x				
Hemodynamic Parameters[6]			x								
**Safety Assessments**											
Telephone Contact		x		x					x		
Concomitant Medication	x	x	x	x	x	x	x	x	x	x	x
Physical Examination	x		x			x	x	x			x
Infusion Site Assessment	x		x[7]		x	x	x	x		x	x
Vital Signs	x		x[7]		x	x	x	x		x	x

		4-week Run-in period	Treatment Phase						Extension Phase		
	**Visit 1**	**Phone call (occurring 14 days prior to baseline)**	**Visit 2**	**Phone call**	**Un-scheduled Visit**	**Early withdraw**	**Visit 3**	**Follow-up Visit[1]**	**Phone call[2] at Month 3 (plus every 6 months)**	**Clinic Visit[3] at Month 6 (plus every 6 month)**	**End of Extension Study Visit or Un-scheduled Visit or Early withdraw**
**Procedures**	**Screen[4]**	**Day-14**	**Baseline[5] (±7 d)**	**Day 3, 5, 7 & Week 2**			**Week 4 (±7 d)**		**Month 3 (±14 d)**	**Month 6 (±14 d)**	
Pulse oximetry (oxygen saturation)	x		x		x	x	x	x		x	x
12-lead ECG	x		x			x	x				
AE/SAE	x	x	x[7]	x	x	x	x	x	x	x	x
**Lab Assessments**											
Clinical Labs (Hematology, Chemistry and Urinalysis)	x		x		x	x	x	x[8]		x	x[8]
Pregnancy Test (urine)	x		x			x	x	x		x	x
NT-ProBNP			x			x	x				
PGx Sampling			x								
**Study Treatment**											
Dispense Study Treatment			x				x			x	
Assess compliance				x	x	x	x		x	x	x

Table 1 Time and Events Table (Continued)

1. Follow-up visit should occur one week after Week 4 visit (Visit 3) for those subjects who are not participating in the extension phase of the study. For those continuing the treatment in the extension, follow up visit will occur at the end of extension study.
2. The first extension telephone call will be scheduled 3 months after baseline. Subsequent telephone calls will be scheduled every 6 months thereafter until the new FLOLAN diluent formulation is commercially available.
3. The first extension clinic visit will be scheduled 6 months after baseline. Subsequent clinic visits will be scheduled every 6 months thereafter until the new FLOLAN diluent formulation is commercially available.
4. Run-in period will start on the day of screening or up to 2 weeks after screening.
5. Subjects will remain in the clinic for a minimum of 6 hours (Day1) post dose or in the hospital for 24 to 48 hours (Days 1 to 2) post dose and then will be discharged from Baseline (Visit 2) per local guidelines and at the discretion of the investigator.
6. Hemodynamic parameters will only be collected at baseline (immediately prior to the switch) and at a single point between 1 to 2 hours after the switch in a subset of subjects where RHC is performed per local practice and per investigator. (see Section 6.1).
7. These safety assessments will be repeated just prior to discharge.
8. Clinical laboratory tests (clinical chemistry, haematology and urinalysis) may be repeated if there were results that were out of reference range from the previous visit or may be performed at the investigator’s discretion.

6.1. Critical Baseline Assessments

Medical and therapy history will be assessed at baseline to ensure that deviations from inclusion/exclusion criteria do not occur. In addition, a six minute walk distance (6MWD) test, Borg dyspnoea index (performed after 6MWD), and WHO functional class (FC) will be assessed.

A subset of subjects will undergo right heart catherisation (RHC) in those sites where this is considered standard of care per local practice and at the discretion of the investigator. Hemodynamic parameters will be collected in this subset at baseline (immediately prior to the switch) and at a single time point between 1 to 2 hours after the switch. Historic hemodynamic data will be obtained, if the subject has had RHC within the last 6 months. The following hemodynamic data will be transcribed into the eCRF: heart rate, mean blood pressure (systolic and diastolic), mean pulmonary arterial pressure in mmHg (mPAP), pulmonary capillary wedge pressure or left ventricular end diastolic pressure, pulmonary vascular resistance (PVR), cardiac index (CI), and right atrial pressure (RAP).

6.2. Efficacy

A six minute walk distance (6MWD) test, Borg dyspnoea index (performed after 6MWD) and QOL assessments using SF-36 questionnaire and a study specific questionnaire will be assessed at baseline and Week 4 or early withdrawal.

WHO functional class (FC) will be assessed at all clinic visits and follow-up visit.

6.3. Safety

6.3.1. Physical examination

Physical examination will be performed at baseline, Week 4 or early withdrawal and at follow-up. Height and weight will also be measured and recorded at the time of physical examination. Infusion site will be inspected for erythema, excoriation, induration, skin necrosis or signs of local sepsis at all clinic visits and at follow-up.

6.3.2. Electrocardiogram

A 12-lead ECG will be performed at baseline and Week 4 or early withdrawal. Any changes since baseline considered to be significant in the medical and scientific judgement of the investigator are to be recorded as AEs or SAEs in the eCRF.

6.3.3. Vital Signs

Vital signs including heart rate and supine blood pressure will be collected at each clinic visit and at follow-up.

6.3.4. Clinical Laboratory Tests

The following tests are required at each clinic visit (including early withdrawal, an unscheduled visit and follow-up [if applicable]).

- **Chemistry:** serum alanine aminotransferase (ALT/SGPT), aspartate aminotransferase (AST/SGOT), alkaline phosphatase, gamma glutamyl transferase (GGT), lactate dehydrogenase (LDH), creatine phosphokinase (CPK), total bilirubin, creatinine, sodium, magnesium, potassium, chloride, bicarbonate (CO2), phosphorus-inorganic, calcium, blood urea nitrogen (BUN), uric acid, glucose, total protein, and albumin, thyroid function test (T3, T4 and TSH)
- **Haematology:** haemoglobin, hematocrit, red cell count, red cell indices (mean corpuscular volume [MCV], mean corpuscular haemoglobin [MCH], and mean corpuscular haemoglobin concentration [MCHC]), white blood cell count (total and differential), reticulocyte count, and platelet count.
- Dipstick urinalysis (to measure blood, glucose and protein).
- **Pregnancy:** Urine pregnancy test will be collected at screening, baseline, Week 4 or early withdrawal, and follow-up.

Blood samples for NT-Pro BNP concentration will only be collected at baseline, and at Week 4 or at early withdrawal (if applicable).

6.3.5. Contraception Requirements

Female subjects of childbearing potential must not become pregnant and so must be sexually inactive by abstinence or use contraceptive methods with a failure rate of < 1%.

Abstinence

Sexual inactivity by abstinence must be consistent with the preferred and usual lifestyle of the subject. Periodic abstinence (e.g. calendar, ovulation, symptothermal, post-ovulation methods) and withdrawal are not acceptable methods of contraception.

Contraceptive Methods with a Failure Rate of < 1%

- Oral contraceptive, either combined or progestogen alone
- Injectable progestogen
- Implants of levonorgestrel
- Estrogenic vaginal ring
- Percutaneous contraceptive patches
- Intrauterine device (IUD) or intrauterine system (IUS) that meets the <1% failure rate as stated in the product label

- Male partner sterilization (vasectomy with documentation of azoospermia) prior to the female subject's entry into the study, and this male is the sole partner for that subject. For this definition, “documented” refers to the outcome of the investigator's/designee’s medical examination of the subject or review of the subject's medical history for study eligibility, as obtained via a verbal interview with the subject or from the subject’s medical records.
- Male condom combined with a vaginal spermicide (foam, gel, film, cream or suppository.
- Male Condom combined with a female diaphragm, either with or without a vaginal spermicide (foam/gel/film/cream/suppository)

These allowed methods of contraception are only effective when used consistently, correctly and in accordance with the product label. The investigator is responsible for ensuring subjects understand how to properly use these methods of contraception.

6.3.6. Adverse Events

The investigator or site staff will be responsible for detecting, documenting and reporting events that meet the definition of an AE or SAE.

6.3.6.1. Definition of an AE

Any untoward medical occurrence in a patient or clinical investigation subject, temporally associated with the use of a medicinal product, whether or not considered related to the medicinal product.

Note: An AE can therefore be any unfavorable and unintended sign (including an abnormal laboratory finding), symptom, or disease (new or exacerbated) temporally associated with the use of a medicinal product. For marketed medicinal products, this also includes failure to produce expected benefits (i.e., lack of efficacy), abuse or misuse.

Planned admission to a hospital setting to facilitate the transition from the current formulation of FLOLAN to the new formulation of FLOLAN as per local guidelines or medical practice and not due to any safety concern for the patient will not be considered adverse events unless an event which meets the criteria for an AE (as defined below under the section “Events meeting the definition of an AE include”) occurs during the hospitalization. If an AE occurs during the hospitalization, reporting of that event should occur per the outlined process in Section 6.3.6.1 and Section 6.3.6.2 of the protocol.

Events meeting the definition of an AE include:

- Exacerbation of a chronic or intermittent pre-existing condition including either an increase in frequency and/or intensity of the condition
- New conditions detected or diagnosed after study treatment administration even though it may have been present prior to the start of the study

- Signs, symptoms, or the clinical sequelae of a suspected interaction
- Signs, symptoms, or the clinical sequelae of a suspected overdose of either study treatment or a concomitant medication (overdose per se will not be reported as an AE/SAE).

"Lack of efficacy" or "failure of expected pharmacological action" per se will not be reported as an AE or SAE. However, the signs and symptoms and/or clinical sequelae resulting from lack of efficacy will be reported if they fulfil the definition of an AE or SAE.

Events that **do not** meet the definition of an AE include:

- Medical or surgical procedure (e.g., endoscopy, appendectomy); the condition that leads to the procedure is an AE
- Situations where an untoward medical occurrence did not occur (social and/or convenience admission to a hospital)
- Anticipated day-to-day fluctuations of pre-existing disease(s) or condition(s) present or detected at the start of the study that do not worsen
- The disease/disorder being studied, or expected progression, signs, or symptoms of the disease/disorder being studied, unless more severe than expected for the subject's condition

6.3.6.2. Definition of a SAE

A serious adverse event is any untoward medical occurrence that, at any dose:

a. Results in death

b. Is life-threatening

   NOTE: The term 'life-threatening' in the definition of 'serious' refers to an event in which the subject was at risk of death at the time of the event. It does not refer to an event, which hypothetically might have caused death, if it were more severe.

c. Requires hospitalization or prolongation of existing hospitalization

   NOTE: In general, hospitalization signifies that the subject has been detained (usually involving at least an overnight stay) at the hospital or emergency ward for observation and/or treatment that would not have been appropriate in the physician's office or out-patient setting. Complications that occur during hospitalization are AEs. If a complication prolongs hospitalization or fulfills any other serious criteria, the event is serious. When in doubt as to whether "hospitalization" occurred or was necessary, the AE should be considered serious.

   Hospitalization for elective treatment of a pre-existing condition that did not worsen from baseline is not considered an AE.

d. Results in disability/incapacity, or

NOTE: The term disability means a substantial disruption of a person's ability to conduct normal life functions. This definition is not intended to include experiences of relatively minor medical significance such as uncomplicated headache, nausea, vomiting, diarrhea, influenza, and accidental trauma (e.g. sprained ankle) which may interfere or prevent everyday life functions but do not constitute a substantial disruption.

e. Is a congenital anomaly/birth defect

f. Medical or scientific judgement should be exercised in deciding whether reporting is appropriate in other situations, such as important medical events that may not be immediately life-threatening or result in death or hospitalization but may jeopardize the subject or may require medical or surgical intervention to prevent one of the other outcomes listed in the above definition. These should also be considered serious. Examples of such events are invasive or malignant cancers, intensive treatment in an emergency room or at home for allergic bronchospasm, blood dyscrasias or convulsions that do not result in hospitalization, or development of drug dependency or drug abuse.

g. All events of possible drug-induced liver injury with hyperbilirubinaemia defined as ALT ≥ 3xULN **and** bilirubin ≥ 2xULN (>35% direct) (or ALT ≥ 3xULN and INR>1.5, if INR measured) termed 'Hy's Law' events (INR measurement is not required and the threshold value stated will not apply to patients receiving anticoagulants).

NOTE: bilirubin fractionation is performed if testing is available. If testing is unavailable, record presence of detectable urinary bilirubin on dipstick indicating direct bilirubin elevations and suggesting liver injury. If testing is unavailable and a subject meets the criterion of total bilirubin ≥ 2xULN, then the event is still reported as an SAE. If INR is obtained, include values on the SAE form. INR elevations >1.5 suggest severe liver injury.

6.3.7. Laboratory and Other Safety Assessment Abnormalities Reported as AEs and SAEs

Any abnormal laboratory test results (hematology, clinical chemistry, or urinalysis) or other safety assessments (e.g., ECGs, radiological scans, vital signs measurements), including those that worsen from baseline, and felt to be clinically significant in the medical and scientific judgement of the investigator are to be recorded as AEs or SAEs.

However, any clinically significant safety assessments that are associated with the underlying disease, unless judged by the investigator to be more severe than expected for the subject's condition, are **not** to be reported as AEs or SAEs.

6.3.8. Pregnancy

Any pregnancy that occurs during study participation must be reported using a clinical trial pregnancy form. To ensure subject safety, each pregnancy must be reported to GSK within 2 weeks of learning of its occurrence. The pregnancy must be followed up to determine outcome (including premature termination) and status of mother and child. Pregnancy complications and elective terminations for medical reasons must be reported as an AE or SAE. Spontaneous abortions must be reported as an SAE.

Any SAE occurring in association with a pregnancy brought to the investigator's attention after the subject has completed the study and considered by the investigator as possibly related to the study treatment, must be promptly reported to GSK.

In addition, the investigator must attempt to collect pregnancy information on any female partners of male study subjects who become pregnant while the subject is enrolled in the study. Pregnancy information must be reported to GSK as described above.

6.3.9. Time Period and Frequency of Detecting AEs and SAEs

The investigator or site staff is responsible for detecting, documenting and reporting events that meet the definition of an AE or SAE.

AEs will be collected from the start of study treatment and until the follow up contact.

SAEs will be collected over the same time period as stated above for AEs. However, any SAEs assessed **as related** to study participation (e.g., study treatment, protocol-mandated procedures, invasive tests, or change in existing therapy) or related to a GSK concomitant medication, will be recorded from the time a subject consents to participate in the study up to and including any follow up contact. All SAEs will be reported to GSK within 24 hours, as indicated in Section 6.3.10.

6.3.10. Prompt Reporting of Serious Adverse Events and Other Events to GSK

SAEs, pregnancies, medical device incidents, and liver function abnormalities meeting pre-defined criteria will be reported promptly by the investigator to GSK as described in the following table once the investigator determines that the event meets the protocol definition for that event.

	Initial Reports		Follow-up Information on a Previous Report	
**Type of Event**	**Time Frame**	**Documents**	**Time Frame**	**Documents**
All SAEs	24 hours	"SAE" data collection tool	24 hours	Updated "SAE" data collection tool
Pregnancy	2 Weeks	Pregnancy Notification Form	2 Weeks	Pregnancy Follow up Form

The method of detecting, recording, evaluating and follow-up of AEs and SAEs plus procedures for completing and transmitting SAE reports to GSK are provided in the SPM. Procedures for post-study AEs/SAEs are provided in the SPM.

6.3.10.1. Regulatory reporting requirements for SAEs

Prompt notification of SAEs by the investigator to GSK is essential so that legal obligations and ethical responsibilities towards the safety of subjects are met.

GSK has a legal responsibility to notify both the local regulatory authority and other regulatory agencies about the safety of a product under clinical investigation. GSK will comply with country specific regulatory requirements relating to safety reporting to the regulatory authority, Institutional Review Board (IRB)/Independent Ethics Committee (IEC) and investigators.

Investigator safety reports are prepared for suspected unexpected serious adverse reactions according to local regulatory requirements and GSK policy and are forwarded to investigators as necessary.

An investigator who receives an investigator safety report describing a SAE(s) or other specific safety information (e.g., summary or listing of SAEs) from GSK will file it with the IB and will notify the IRB/IEC, if appropriate according to local requirements.

6.4. Pharmacogenetics

Information regarding pharmacogenetic research is included in Appendix 1. The IEC/IRB and, where required, the applicable regulatory agency must approve the PGx assessments before these can be conducted at the site. The approval(s) must be in writing and will clearly specify approval of the PGx assessments. In some cases, approval of the PGx assessments can occur after approval is obtained for the rest of the study. If so, then the written approval will clearly indicate approval of the PGx assessments is being deferred and the study, except for PGx assessments, can be initiated. When PGx assessments will not be approved, then the approval for the rest of the study will clearly indicate this and therefore, PGx assessments will not be conducted.

7. DATA MANAGEMENT

For this study, subject data will be entered into GSK defined electronic case report forms (eCRFs), transmitted electronically to GSK and combined with data provided from other sources in a validated data system.

Management of clinical data will be performed in accordance with applicable GSK standards and data cleaning procedures to ensure the integrity of the data, e.g., removing errors and inconsistencies in the data. Adverse events and concomitant medications terms will be coded using MedDRA and an internal validated medication dictionary, GSKDrug. An appropriate medical dictionary that covers all approved drugs in the region will be referenced. eCRFs (including queries and audit trails) will be retained by GSK, and copies will be sent to the investigator to maintain as the investigator copy. In all cases, subject initials will not be collected or transmitted to GSK according to GSK policy.

8. DATA ANALYSIS AND STATISTICAL CONSIDERATIONS

8.1. Hypotheses

Summary displays will be limited to descriptive statistics. No formal hypothesis tests are planned.

8.2. Study Design Considerations

8.2.1. Sample Size Assumptions

The sample size for this study is based on feasibility without formal power calculations to justify sample size. Formal hypothesis testing is not planned. The safety and efficacy data will be summarized and precision quantified using 95% confidence intervals.

8.2.2. Sample Size Sensitivity

The sample size for this study is based on feasibility.

8.2.3. Sample Size Re-estimation

No sample size re-estimation is planned.

8.3. Data Analysis Considerations

8.3.1. Analysis Populations

The Intention-to-Treat (ITT) population will consist of all subjects who received at least 1 dose of study drug. The ITT population will be used for all efficacy and safety summaries.

Subjects will be excluded from the analysis populations if they fell to take at least one dose of study treatment.

8.3.2. Analysis Data Sets

Analysis datasets will consist of all data collected in the study and evaluated according to the population described in Section 8.3.1.

8.3.3. Treatment Comparisons

No formal comparisons are planned for this study.

8.3.3.1. Primary Measurements of Interest

The primary measurements of interest will be the following:

- Quality of life estimates, based on the SF-36 questionnaire
- Ease of administration and changes in quality of life, in particular activities of daily living, as assessed by using study specific questionnaire
- Dose titration, measured by changes from baseline in the dose of thermo stable FLOLAN formulation at the time of discharge (at minimum of 6 hours or per local guidelines/practices)

8.3.3.2. Secondary Measurements of Interest

The secondary measurements of interest are the following:

- Hemodynamic parameters (Mean Pulmonary artery blood pressure in mmHg [mPAP], Right Vascular resistance [PVR], Cardiac Index [CI], Right Atrial Pressure [RAP].
- Six minute walking distance (6MWD)
- Breathlessness after 6MWD – Borg Dyspnoea index
- Who functional class
- Adverse events and Serious adverse events
- Vital signs: systolic and diastolic blood pressure, heart rate
- Clinical laboratory tests (clinical chemistry, hematology, and Urinalysis)
- 12-lead ECG
- Oxygen saturation
- Infusion site reactions including erythema, excoriation, induration, skin necrosis, or signs of local sepsis
- Urine pregnancy test

All data will be presented in total for the whole treatment period or by visit, where available.

8.3.3.3. Other Measurements of Interest

No other measurements of interest are planned.

8.3.4. Interim Analysis

An interim analysis is not planned.

8.3.5. Key Elements of Analysis Plan

The Analysis Plan will consist of summary and graphical displays. Individual patient listings will also be included. Full details will be described in a "Reporting and Analysis Plan" that is authored in accordance with SOP_54838 v4 Development, Review and Approval of Reporting and Analysis Plan.s

8.3.5.1. Quality of life analysis

Quality of life data will be presented as number and percentage of subject in each answer category for a particular question, as well as overall score. Data will be presented for each visit (Baseline, end of 4-week treatment period).

8.3.5.2. Safety Analyses

All subjects who received at least one dose of study medication will be assessed for clinical safety and tolerability.

Clinical interpretation will be based upon review of displays of adverse events, laboratory values (hematology, chemistry, urinalysis), vital signs, 12-lead ECG, pulse oximetry, NT-ProBNP, physical examination, infusion site assessment, and pregnancy tests. Principal considerations in this evaluation of adverse events will be investigator-reported relationship of either adverse events or laboratory abnormalities to study medication. For each laboratory test, the number and percentage of subjects with values above the reference range will be displayed for each treatment group and overall. Appropriate monitoring of safety data will be conducted throughout the conduct of the study.

No formal statistical hypothesis testing is planned for safety parameters. All data will be presented descriptively or graphically.

8.3.5.3. Efficacy Analyses

No formal efficacy analyses are planned. Descriptive statistics on observed values and change from screening (standard formulation) or baseline (new formulation) will be provided.

8.3.5.4. Other Analyses

All other data (demography, medical history, disease history, therapy history, concomitant medications, exposure, compliance to study drug, infusion site assessment) will be summarized descriptively, and by visit, where available.

8.3.5.5. Pharmacogenetic Analyses

Pharmacogenetic analysis is not currently planned for this study. However, if at any time it appears that there is a potential unexpected or unexplained variation in drug handling or response (e.g., pharmacodynamic, efficacy and/or safety) that may be attributable to

genetic variation, then PGx analysis may be conducted. In these circumstances the analysis undertaken will be limited to PGx analysis of response to or handling of the new thermo stable FLOLAN.

9. STUDY CONDUCT CONSIDERATIONS

9.1. Posting of Information on Clinicaltrials.gov

Study information from this protocol will be posted on clinicaltrials.gov before enrolment of subjects begins.

9.2. Regulatory and Ethical Considerations, Including the Informed Consent Process

Prior to initiation of a study site, GSK will obtain approval from the appropriate regulatory agency to conduct the study in accordance with ICH Good Clinical Practice (GCP) and applicable country-specific regulatory requirements.

The study will be conducted in accordance with all applicable regulatory requirements.

The study will be conducted in accordance with ICH GCP, all applicable subject privacy requirements, and the ethical principles that are outlined in the Declaration of Helsinki 2008, including, but not limited to:

- Institutional Review Board (IRB)/Independent Ethics Committee (IEC) review and approval of study protocol and any subsequent amendments.
- Subject informed consent.
- Investigator reporting requirements.

GSK will provide full details of the above procedures, either verbally, in writing, or both.

Written informed consent must be obtained from each subject prior to participation in the study.

9.3. Quality Control (Study Monitoring)

In accordance with applicable regulations, GCP, and GSK procedures, GSK monitors will contact the site prior to the start of the study to review with the site staff the protocol, study requirements, and their responsibilities to satisfy regulatory, ethical, and GSK requirements. When reviewing data collection procedures, the discussion will include identification, agreement and documentation of data items for which the CRF will serve as the source document.

GSK will monitor the study to ensure that the:

- Data are authentic, accurate, and complete.
- Safety and rights of subjects are being protected.
- Study is conducted in accordance with the currently approved protocol and any other study agreements, GCP, and all applicable regulatory requirements.

The investigator and the head of the medical institution (where applicable) agrees to allow the monitor direct access to all relevant documents.

9.4. Quality Assurance

To ensure compliance with GCP and all applicable regulatory requirements, GSK may conduct a quality assurance audit of the site records, and the regulatory agencies may conduct a regulatory inspection at any time during or after completion of the study. In the event of an audit or inspection, the investigator (and institution) must agree to grant the auditor(s) and inspector(s) direct access to all relevant documents and to allocate their time and the time of their staff to discuss any findings/relevant issues.

9.5. Study and Site Closure

Upon completion or termination of the study, the GSK monitor will conduct site closure activities with the investigator or site staff (as appropriate), in accordance with applicable regulations, GCP, and GSK Standard Operating Procedures.

GSK reserves the right to temporarily suspend or terminate the study at any time for reasons including (but not limited to) safety issues, ethical issues, or severe non-compliance. If GSK determines that such action is required, GSK will discuss the reasons for taking such action with the investigator or head of the medical institution (where applicable). When feasible, GSK will provide advance notice to the investigator or head of the medical institution of the impending action.

If a study is suspended or terminated for **safety reasons**, GSK will promptly inform all investigators, heads of the medical institutions (where applicable),and/or institutions conducting the study. GSK will also promptly inform the relevant regulatory authorities of the suspension/termination along with the reasons for such action. Where required by applicable regulations, the investigator or head of the medical institution must inform the IRB/IEC promptly and provide the reason(s) for the suspension/termination.

9.6. Records Retention

Following closure of the study, the investigator or head of the medical institution (where applicable) must maintain all site study records (except for those required by local regulations to be maintained elsewhere) in a safe and secure location. The records must be easily accessible when needed (e.g., for a GSK audit or regulatory inspection) and must be available for review in conjunction with assessment of the facility, supporting systems, and relevant site staff.

Where permitted by local laws/regulations or institutional policy, some or all of the records may be maintained in a format other than hard copy (e.g., microfiche, scanned, electronic); however, caution must be exercised before such action is taken. The investigator must ensure that all reproductions are legible and are a true and accurate copy of the original. In addition, they must meet accessibility and retrieval standards, including regeneration of a hard copy, if required. The investigator must also ensure that an acceptable back-up of the reproductions exists and that there is an acceptable quality control procedure in place for creating the reproductions.

GSK will inform the investigator of the time period for retaining the site records in order to comply with all applicable regulatory requirements. The minimum retention time will meet the strictest standard applicable to a particular site, as dictated by local laws/regulations, GSK standard operating procedures, and/or institutional requirements.

The investigator must notify GSK of any changes in the archival arrangements, including, but not limited to archival of records at an off-site facility or transfer of ownership of the records in the event that the investigator is no longer associated with the site.

9.7. Provision of Study Results to Investigators, Posting to the Clinical Trials Register and Publication

Where required by applicable regulatory requirements, an investigator signatory will be identified for the approval of the clinical study report. The investigator will be provided reasonable access to statistical tables, figures, and relevant reports and will have the opportunity to review the complete study results at a GSK site or other mutually-agreeable location.

GSK will also provide the investigator with the full summary of the study results. The investigator is encouraged to share the summary results with the study subjects, as appropriate.

The results summary will be posted to the Clinical Study Register at the time of the first regulatory approval or within 12 months of any decision to terminate development. In addition, a manuscript will be submitted to a peer-reviewed journal for publication within 12 months of the first approval or within 12 months of any decision to terminate development. When manuscript publication in a peer-reviewed journal is not feasible, further study information will be posted to the GSK Clinical Study Register to supplement the results summary.

The results summary will be posted to the Clinical Study Register no later than 12 months after the last subject's last visit (LSLV) or sooner if required by legal agreement, local law or regulation. In addition, a manuscript will be submitted to a peer-reviewed journal for publication within 18 months of LSLV. When manuscript publication in a peer-reviewed journal is not feasible, further study information will be posted to the GSK Clinical Study Register to supplement the results summary.

A manuscript will be progressed for publication in the scientific literature if the results provide important scientific or medical knowledge.

9.8. Independent Data Monitoring Committee (IDMC)

An IDMC will be not be utilized in this study.

10. REFERENCES

Galie N, Torbicki A, Barst B, Dartevelle P, Haworth S, Higenbottam T, et al. Guidelines on diagnosis and treatment of pulmonary arterial hypertension. The Task Force on Diagnosis and Treatment of Pulmonary Arterial Hypertension of the European Society of Cardiology. Eur Heart J. 2004;25:2243–78.

Horn EM, Barst RJ. Treprostinil therapy for pulmonary artery hypertension. Expert Opin Investig Drugs. 2002;11(11):1615-22.

Humbert M, Morrell NW, Archer SL, Stenmark KR, MacLean MR, Lang M. Cellular and molecular pathobiology of pulmonary arterial hypertension. J Amer Coll Cardiol. 2004;43(12 Suppl S):13S-24S.

Jones DA, Benjamin CW, Linseman DA. Activation of thromboxane and prostacyclin receptors elicits opposing effects on vascular smooth muscle cell growth and mitogen-activated protein kinase signaling cascades. Mol Pharmacol. 1995;48:890–6.

McLaughlin VV, Shillington A, Rich S. Survival in primary pulmonary hypertension: the impact of epoprostenol therapy. Circulation. 2002;106:1477–82.

Simonneau G, Barst RJ, Galie N, Naeije R, Rich S, Bourge R. Continuous subcutaneous infusion of treprostinil, a prostacyclin analogue, in patients with pulmonary arterial hypertension. A double-blind, randomized, placebo-controlled trial. Am J Respir Crit Care Med 2002;165:800–4.

Sitbon O, Humbert M, Nunes H, Parent F, Garcia G, Herve G. Long-term intravenous epoprostenol infusion in primary pulmonary hypertension: prognostic factors and survival. J Am Coll Cardiol. 2002;40:780–8.

11. APPENDICES

11.1. Appendix 1: PGx

Pharmacogenetic Research

Pharmacogenetics – Background

Pharmacogenetics (PGx) is the study of variability in drug response due to hereditary factors in different populations. There is increasing evidence that an individual's genetic composition (i.e., genotype) may impact the pharmacokinetics (absorption, distribution, metabolism, elimination), pharmacodynamics (relationship between concentrations and pharmacologic effects or the time course of pharmacologic effects) and/or clinical outcome (in terms of efficacy and/or safety and tolerability). Some reported examples of PGx analysis include:

Drug	Disease	Gene	Outcome
Abacavir	HIV [Hetherington, 2002; Mallal, 2002]	HLA –B*5701	Individuals with HLA-B*5701 variant may be at increased risk for experiencing hypersensitivity to abacavir. Clinical assays are available for HLA-B*5701 but none has been validated. HLA-B*5701 screening would supplement but never replace abacavir clinical risk management strategies aimed at minimising rare but serious outcomes associated with abacavir hypersensitivity.
Warfarin	Cardiovascular [Neergard, 2006; Wilke, 2005]	CYP2C9	Serious adverse events (SAEs) experienced by some patients on warfarin may be explained by variations in the CYP2C9 gene that influences the degree of anticoagulation achieved.
Irinotecan	Cancer [FDA News Release, 2005]	UGT1A1	Variations in the UGT1A1 gene can influence a patient's ability to break down irinotecan, which can lead to increased blood levels of the drug and a higher risk of side effects. A dose of irinotecan that is safe for one patient with a particular UGT1A1 gene variation, might be too high for another patient without this variation, raising the risk of certain side-effects.<br>A genetic blood test (Invader UGT1A1 molecular assay) is available that can detect variations in the gene.

A key component to successful PGx research is the collection of samples during the conduct of clinical studies.

Collection of whole blood samples, even when no *a priori* hypothesis has been identified, may enable PGx analysis to be conducted if at any time it appears that there is a potential unexpected or unexplained variation in handling or response to FLOLAN.

Pharmacogenetic Research Objectives

The objective of the PGx research (if there is a potential unexpected or unexplained variation) is to investigate a possible genetic relationship to handling or response to FLOLAN. If at any time it appears there is potential variability in response in this clinical study or in a series of clinical studies with FLOLAN and the new diluent reformulation that may be attributable to genetic variations of subjects, the following objectives may be investigated:

- Relationship between genetic variants and the pharmacokinetics and/or pharmacodynamics of study treatment
- Relationship between genetic variants and safety and/or tolerability of study treatment
- Relationship between genetic variants and efficacy of study treatment

Study Population

Any subject who has given informed consent to participate in the clinical study, has met all the entry criteria for the clinical study, and receives study treatment may take part in the PGx research. Any subject who has received an allogeneic bone marrow transplant must be excluded from the PGx research.

Subject participation in the PGx research is voluntary and refusal to participate will not indicate withdrawal from the clinical study. Refusal to participate will involve no penalty or loss of benefits to which the subject would otherwise be entitled.

Study Assessments and Procedures

Blood or saliva samples can be taken for PGx assessments.

If taking blood samples: in addition to any blood samples taken for the clinical study, a whole blood sample (~10ml) will be collected for the PGx research using a tube containing EDTA. It is recommended that the blood sample be taken at the first opportunity after a subject has been entered and provided informed consent for PGx research, but may be taken at any time while the subject is participating in the clinical study.

If taking saliva collections, no additional whole blood samples will be necessary for the PGx analysis. Deoxyribonucleic acid (DNA) will be extracted from cells obtained from the subject's saliva. Saliva (2mL) is spit into the DNA self-collection kit. A single sample will be taken but can be duplicated if the first sample is unusable. It is recommended that the saliva sample be collected at baseline.

The PGx sample is labelled (or "coded") with a study specific number that can be traced or linked back to the subject by the investigator or site staff. Coded samples do not carry personal identifiers (such as name or social security number). The blood/saliva sample is taken on a single occasion unless a duplicate sample is required due to inability to utilize the original sample.

The DNA extracted from the blood/saliva sample may be subjected to sample quality control analysis. This analysis will involve the genotyping of several genetic markers to confirm the integrity of individual samples. If inconsistencies are noted in the analysis, then those samples may be destroyed.

The need to conduct PGx analysis may be identified after a study (or a set of studies) of FLOLAN has been completed and the study data reviewed.

In some cases, the samples may not be studied. e.g., no questions are raised about how people respond to FLOLAN.

Samples will be stored securely and may be kept for up to 15 years after the last subject completes the study or GSK may destroy the samples sooner. GSK or those working with GSK (for example, other researchers) will use samples collected from the study for the purpose stated in this protocol and in the informed consent form.

Subjects can request their sample to be destroyed at any time.

Subject Withdrawal from Study

If a subject who has consented to participate in PGx research and has a sample taken for PGx research withdraws from the clinical study for any reason other than lost to follow-up, the subject will be given the following options:

- The sample is retained for PGx research
- Any PGx sample is destroyed.

If a subject withdraws consent from the PGx research or requests sample destruction for any reason, the investigator must complete the appropriate documentation to request sample destruction within the timeframe specified by GSK and maintain the documentation in the site study records. If the sample has already been processed, it will be destroyed after all steps are complete. GSK will ensure that any data related to the sample will not be analysed. The sample will be destroyed after processing is complete.

Screen and Baseline Failures

If a blood sample for PGx research has been collected and it is determined that the subject does not meet the entry criteria for participation in the clinical study, then the investigator must complete the appropriate documentation to request sample destruction within 5 days. The sample will be destroyed and documentation sent to the site within 30 working days of receipt of the request for destruction. All documents pertaining to sample destruction must be maintained in the site study records.

Pharmacogenetics Analyses

1. Specific sections of DNA may be selected from areas of the genome (e.g., candidate genes) known to encode the drug target, drug metabolizing enzymes, areas associated with mechanisms underlying adverse events, and those linked to study disease and, thus, linked to drug response.

The candidate genes that may be investigated in this study include the following: the GSK Absorption, Distribution, Metabolism and Excretion genes. These play a central role in drug pharmacokinetics and pharmacodynamics. In addition, continuing research may identify other enzymes, transporters, proteins or receptors that may be involved in response to FLOLAN. The genes that may code for these proteins may also be studied.

2. By evaluating large numbers of polymorphic markers (e.g., single nucleotide polymorphisms or SNPs) throughout the genome, sets of markers may be identified that correspond to differential drug response.

The results of PGx investigations will be reported either as part of the main clinical study report or as a separate report. All endpoints of interest from all comparisons will be descriptively and/or graphically summarised as appropriate to the data. In all cases, appropriate statistical methods will be used to analyse the genetic markers in the context of other clinical data. Statistical methods may include, but are not limited to Hardy-Weinberg Equilibrium testing, Comparison of Demographic and Baseline Characteristics by Genotype, Evaluation of Genotypic Effects, Evaluation of Treatment by Genotype and Gene-Gene Interaction, Linkage Disequilibrium, Multiple Comparison and Multiplicity and/or Power and Sample Size Considerations. Detailed description of the analyses to be conducted will be documented in the Pharmacogenetics Reporting and Analysis Plan.

3. Genome-wide scans involving a large number of polymorphic markers (e.g., single nucleotide polymorphisms) located throughout the genome. This approach is often employed when potential genetic effects are not well understood.

Informed Consent

Subjects who do not wish to participate in the PGx research may still participate in the clinical study. PGx informed consent must be obtained prior to any blood/saliva being taken for PGx research.

Provision of Study Results and Confidentiality of Subject's PGx Data

GSK may summarize the cumulative PGx research results in the clinical study report.

In general, GSK does not inform the investigator, subject, or anyone else (e.g., family members, study investigators, primary care physicians, insurers, or employers) of the PGx research results that are not known to be relevant to the subject's medical care at the time of the study, because the information generated from PGx studies is preliminary in nature, and the significance and scientific validity of the results are undetermined at such an early stage of research, under any circumstances unless required by law.

References

Hetherington S, Hughes AR, Mosteller M, Shortino D, Baker KL, Spreen W, Lai E, Davies K, Handley A, Dow DJ, Fling ME, Stocum M, Bowman C, Thurmond LM, Roses AD. Genetic variations in HLA-B region and hypersensitivity reactions to abacavir. Lancet. 2002; 359:1121-2.

Mallal S, Nolan D, Witt C, Masel G, Martin AM, Moore C, Sayer D, Castley A, Mamotte C, Maxwell D, James I. Association between presence of HLA-B*5701, HLA-DR7, and HLA-DQ3 and hypersensitivity to HIV-1 reverse-transcriptase inhibitor abacavir. Lancet. 2002; 359:727-32.

Neergard. Reducing the risk of blood thinners. Associated press, September 2006.

U.S. Food and Drug Administration, FDA Clears Genetic Test That Advances Personalized Medicine Test Helps Determine Safety of Drug Therapy 22 August 2005, http://www.fda.gov/bbs/topics/NEWS/2005/NEW01220.html.

Wilke RA, Musana AK, Weber WW. Cytochrome P450 gene-based drug prescribing, and factors impacting translation into routine clinical practice. Personalized Med 2005; 2: 213-224.

11.2. Appendix 2: Protocol Changes

Changes Resulting From Protocol Amendment 01

This amendment is applicable to all sites.

Summary of Changes

1. Correction of the IND number and addition of the EudraCT Number to the Sponsor Information Page.
2. Removal of extraneous wording in Section 6.3.6.1 Definition of an AE, which was included in error and conflicts with other wording in this section.
3. Addition of dipstick urinalysis to Section 6.3.4 Clinical Laboratory Tests which was inadvertently missed out of this section. This will provide consistency with the Time and Events Table.
4. Correction to the spelling of urinalysis.

List of Specific Changes

Sponsor Information Page

Original Text:

Regulatory Agency Identifying Number(s): IND 016549;

Amended Text:

Regulatory Agency Identifying Number(s): IND 016459;
EudraCT Number 2011-002943-92

Protocol Summary, Secondary Endpoints, bullet 4; Section 8.3.3.2 Secondary Measurements of Interest and 8.3.5.2 Safety Analyses

Original Text:

Clinical laboratory tests (clinical chemistry, hematology and urinanalysis)

Amended Text:

Clinical laboratory tests (clinical chemistry, hematology and urinalysis)

Section 6.3.4 Clinical Laboratory Tests

Original Text:

None.

Amended Text:

Addition of a new bullet 3:

Dipstick urinalysis (to measure blood, glucose and protein).

<u>Section 6.3.6.1 Definition of an AE, Paragraph 5 and 6</u>

Original Text:

"Lack of efficacy" or "failure of expected pharmacological action" per se will not be reported as an AE or SAE. However, the signs and symptoms and/or clinical sequelae resulting from lack of efficacy will be reported if they fulfil the definition of an AE or SAE.

The signs and symptoms and/or clinical sequelae resulting from lack of efficacy will be reported if they fulfill the definition of an AE or SAE. Also, "lack of efficacy" or "failure of expected pharmacological action" also constitutes an AE or SAE.

Amended Text:

"Lack of efficacy" or "failure of expected pharmacological action" per se will not be reported as an AE or SAE. However, the signs and symptoms and/or clinical sequelae resulting from lack of efficacy will be reported if they fulfil the definition of an AE or SAE.

~~The signs and symptoms and/or clinical sequelae resulting from lack of efficacy will be reported if they fulfill the definition of an AE or SAE. Also, "lack of efficacy" or "failure of expected pharmacological action" also constitutes an AE or SAE.~~
