# Supplementary material for: Quality of Life, Safety and Efficacy Profile of Thermostable Flolan in Pulmonary Arterial Hypertension
Source: PLoS One. 2015 Mar 20;10(3):e0120657. doi: 10.1371/journal.pone.0120657 (PMC4368561; doi:10.1371/journal.pone.0120657)
Supplement: S1 CONSORT Checklist — (PDF) [file pone.0120657.s002.pdf]

TREND Statement Checklist

Paper Section/ Topic	Item No	Descriptor	Reported? ✓	Pg #
**Title and Abstract**				
Title and Abstract	1	• Information on how unit were allocated to interventions	Reported	Abstract
		• Structured abstract recommended	Reported	Abstract
		• Information on target population or study sample	Reported	Abstract
**Introduction**				
Background	2	• Scientific background and explanation of rationale	Reported	Introduction
		• Theories used in designing behavioral interventions	Not applicable	
**Methods**				
Participants	3	• Eligibility criteria for participants, including criteria at different levels in recruitment/sampling plan (e.g., cities, clinics, subjects)	Reported	Patients
		• Method of recruitment (e.g., referral, self-selection), including the sampling method if a systematic sampling plan was implemented	Reported	Patients
		• Recruitment setting	Reported	Ethics Statement
		• Settings and locations where the data were collected	Reported	Ethics Statement
Interventions	4	• Details of the interventions intended for each study condition and how and when they were actually administered, specifically including:	Reported	
		○ Content: what was given?	Reported	Procedures
		○ Delivery method: how was the content given?	Reported	Procedures
		○ Unit of delivery: how were the subjects grouped during delivery?	Reported	Procedures
		○ Deliverer: who delivered the intervention?	Reported	Procedures
		○ Setting: where was the intervention delivered?	Reported	Procedures
		○ Exposure quantity and duration: how many sessions or episodes or events were intended to be delivered? How long were they intended to last?	Reported	Procedures
		○ Time span: how long was it intended to take to deliver the intervention to each unit?	Reported	Procedures
		○ Activities to increase compliance or adherence (e.g., incentives)	Not applicable	
Objectives	5	• Specific objectives and hypotheses	Reported	Introduction
Outcomes	6	• Clearly defined primary and secondary outcome measures	Reported	Procedures
		• Methods used to collect data and any methods used to enhance the quality of measurements	Reported	Procedures
		• Information on validated instruments such as psychometric and biometric properties	Reported	Procedures
Sample Size	7	• How sample size was determined and, when applicable, explanation of any interim analyses and stopping rules	Reported	Statistical Analysis
Assignment Method	8	• Unit of assignment (the unit being assigned to study condition, e.g., individual, group, community)	Not applicable Single-arm	
		• Method used to assign units to study conditions, including details of any restriction (e.g., blocking, stratification, minimization)	Not applicable Single-arm	
		• Inclusion of aspects employed to help minimize potential bias induced due to non-randomization (e.g., matching)	Not applicable Single-arm	

TREND Statement Checklist

Blinding (masking)	9	• Whether or not participants, those administering the interventions, and those assessing the outcomes were blinded to study condition assignment; if so, statement regarding how the blinding was accomplished and how it was assessed.	Not applicable Open label	
Unit of Analysis	10	• Description of the smallest unit that is being analyzed to assess intervention effects (e.g., individual, group, or community)	Reported	Statistical Analysis
		• If the unit of analysis differs from the unit of assignment, the analytical method used to account for this (e.g., adjusting the standard error estimates by the design effect or using multilevel analysis)	Unit of analysis = Unit of assignment	
Statistical Methods	11	• Statistical methods used to compare study groups for primary methods outcome(s), including complex methods of correlated data	Statistical methods reported	Statistical Analysis
		• Statistical methods used for additional analyses, such as a subgroup analyses and adjusted analysis	Not applicable	
		• Methods for imputing missing data, if used	Reported	Statistical Analysis
		• Statistical software or programs used	Not reported	
**Results**				
Participant flow	12	• Flow of participants through each stage of the study: enrollment, assignment, allocation, and intervention exposure, follow-up, analysis (a diagram is strongly recommended)	Reported	Patients' disposition and Figure 1
		○ Enrollment: the numbers of participants screened for eligibility, found to be eligible or not eligible, declined to be enrolled, and enrolled in the study	Reported	Patients' disposition and Figure 1
		○ Assignment: the numbers of participants assigned to a study condition	Reported	Patients' disposition and Figure 1
		○ Allocation and intervention exposure: the number of participants assigned to each study condition and the number of participants who received each intervention	Reported	Patients' disposition and Figure 1
		○ Follow-up: the number of participants who completed the follow-up or did not complete the follow-up (i.e., lost to follow-up), by study condition	Reported	Patients' disposition and Figure 1
		○ Analysis: the number of participants included in or excluded from the main analysis, by study condition	Reported	Patients' disposition and Figure 1
		• Description of protocol deviations from study as planned, along with reasons	Reported	Patients' disposition and Figure 1
Recruitment	13	• Dates defining the periods of recruitment and follow-up	Reported	Procedures
Baseline Data	14	• Baseline demographic and clinical characteristics of participants in each study condition	Reported	Table 1
		• Baseline characteristics for each study condition relevant to specific disease prevention research	Not applicable	
		• Baseline comparisons of those lost to follow-up and those retained, overall and by study condition	Not reported	
		• Comparison between study population at baseline and target population of interest	Reported	Figure 2 Figure 3 Figure 4
Baseline equivalence	15	• Data on study group equivalence at baseline and statistical methods used to control for baseline differences	Not applicable	

TREND Statement Checklist

Numbers analyzed	16	• Number of participants (denominator) included in each analysis for each study condition, particularly when the denominators change for different outcomes; statement of the results in absolute numbers when feasible	Reported	Patients' disposition
		• Indication of whether the analysis strategy was "intention to treat" or, if not, description of how non-compliers were treated in the analyses	Reported	Statistical Analysis
Outcomes and estimation	17	• For each primary and secondary outcome, a summary of results for each estimation study condition, and the estimated effect size and a confidence interval to indicate the precision	Reported	QoL and ease of administration assessment. Secondary and exploratory endpoints. Safety.
		• Inclusion of null and negative findings	Not applicable	
		• Inclusion of results from testing pre-specified causal pathways through which the intervention was intended to operate, if any	Not applicable	
Ancillary analyses	18	• Summary of other analyses performed, including subgroup or restricted analyses, indicating which are pre-specified or exploratory	Not applicable	
Adverse events	19	• Summary of all important adverse events or unintended effects in each study condition (including summary measures, effect size estimates, and confidence intervals)	Not applicable	
**DISCUSSION**				
Interpretation	20	• Interpretation of the results, taking into account study hypotheses, sources of potential bias, imprecision of measures, multiplicative analyses, and other limitations or weaknesses of the study	Reported	Discussion
		• Discussion of results taking into account the mechanism by which the intervention was intended to work (causal pathways) or alternative mechanisms or explanations	Reported	Discussion
		• Discussion of the success of and barriers to implementing the intervention, fidelity of implementation	Reported	Discussion
		• Discussion of research, programmatic, or policy implications	Reported	Discussion
Generalizability	21	• Generalizability (external validity) of the trial findings, taking into account the study population, the characteristics of the intervention, length of follow-up, incentives, compliance rates, specific sites/settings involved in the study, and other contextual issues	Not reported	
Overall Evidence	22	• General interpretation of the results in the context of current evidence and current theory	Reported	Conclusion

From: Des Jarlais, D. C., Lyles, C., Crepaz, N., & the Trend Group (2004). Improving the reporting quality of nonrandomized evaluations of behavioral and public health interventions: The TREND statement. *American Journal of Public Health*, 94, 361-366. For more information, visit: http://www.cdc.gov/trendstatement/
