# Supplementary material for: Quality of Life, Safety and Efficacy Profile of Thermostable Flolan in Pulmonary Arterial Hypertension
Source: PLoS One. 2015 Mar 20;10(3):e0120657. doi: 10.1371/journal.pone.0120657 (PMC4368561; doi:10.1371/journal.pone.0120657)
Supplement: S1 QoLassessment — (DOCX) [file pone.0120657.s003.docx]

**S1 QoLassessment:**

**Quality of Life Assessment (Study-Specific questionnaire)**

Ease of administration and changes in QoL, in particular activities of daily living

assessment, using a study-specific questionnaire was a primary endpoint for this study,

which was completed at Baseline (Visit 2) and Week 4 (Visit 3). The study-specific

questionnaire comprised the following 15 questions:

1. How much time on average in a week is required for mixing, loading and attaching

your FLOLAN cassette to the pump? Responses: less than 1 hour; 1 hour to 1 hour

30 minutes; 1 hour 30 minutes to 2 hours; 2 hours to 2 hours 30 minutes; other.

How much do you think that the pump and other items related to your FLOLAN

treatment interfere with the following activities:

2. The ability to perform physical activities (exercising, walking, etc.)? Rated from

1 (not restrictive) to 10 (extremely restrictive).

3. The ability to perform your basic daily activities (shopping, personal care, etc.)?

Rated from 1 (not restrictive) to 10 (extremely restrictive).

4. The ability to perform activities with your family (playing with your children, school

activities, taking children to school, etc.)? Rated from 1 (not restrictive) to

10 (extremely restrictive).

5. The ability to participate in social activities (leisure)? Rated from 1 (not restrictive)

to 10 (extremely restrictive).

6. How comfortable are you with your ability to comply with your FLOLAN treatment

regimen around other activities (i.e., while travelling, working, on holidays

[vacations] etc.)? Rated from 1 (not comfortable) to 10 (extremely comfortable).

7. If you were asked to perform a new activity such as joining a walking group, or

taking up a new hobby, etc., how likely would you be to try? Rated from 1 (not

likely) to 10 (very likely).

8. What is your overall satisfaction with your ability to perform everyday activities?

Rated from 1 (not satisfied) to 10 (extremely satisfied).

Please rate how the FLOLAN treatment regimen, including the pump and related items,

affects your lifestyle on each of the following items:

9. I feel interested in engaging in physical activity. Rated from 1 (do not agree) to 10

(strongly agree).

10. I feel physically restricted from participating in activities due to the demands of the

treatment regimen. Rated from 1 (do not agree) to 10 (strongly agree).

11. I am confident in my ability to take on any new activities. Rated from 1 (do not

agree) to 10 (strongly agree).

12. My FLOLAN treatment regimen constantly weighs on my mind. Rated from 1 (do

not agree) to 10 (strongly agree).

The following questions were asked at Week 4 (Visit 3) only:

13. Within the past seven days, on how many days did you reconstitute your FLOLAN

solution? Responses: 1 to 7 days.

14. In your opinion, what would be the ideal frequency to reconstitute FLOLAN

solution? Responses: daily; every second day; every third day; every fourth day;

every fifth day.

15. Which FLOLAN product do you prefer to use? Responses: the original (Baseline)

product; the new product; I have no preference.
